# Unravelling a trichloroacetic acid-catalyzed cascade access to benzo[*f*]chromeno[2,3-*h*]quinoxalinoporphyrins

**DOI:** 10.3762/bjoc.19.89

**Published:** 2023-08-11

**Authors:** Chandra Sekhar Tekuri, Pargat Singh, Mahendra Nath

**Affiliations:** 1 Department of Chemistry, Faculty of Science, University of Delhi, Delhi 110 007, India

**Keywords:** bathochromic shift, benzo[*f*]chromeno[2,3-*h*]quinoxalinoporphyrins, catalysis, multicomponent synthesis, one-pot reaction, trichloroacetic acid

## Abstract

A facile one-pot four-component synthetic methodology is evolved to construct novel copper(II) benzo[*f*]chromeno[2,3-*h*]quinoxalinoporphyrins in good yields via a sequential reaction of copper(II) 2,3-diamino-5,10,15,20-tetraarylporphyrins, 2-hydroxynaphthalene-1,4-dione, aromatic aldehydes, and dimedone in the presence of a catalytic amount of trichloroacetic acid in chloroform at 65 °C. Further, the newly prepared copper(II) porphyrins were transformed to the corresponding free base and zinc(II) benzo[*f*]chromeno[2,3-*h*]quinoxalinoporphyrins under standard demetallation and zinc insertion conditions. The absorption and emission properties of the obtained porphyrins were investigated by using UV–visible and fluorescence spectroscopy. The preliminary photophysical results revealed a significant red-shift in their absorption and emission spectra as compared to the *meso*-tetrakis(4-methylphenyl)porphyrins due to the extended π-conjugation.

## Introduction

π-Conjugated porphyrin macrocycles are known for their applications in numerous areas ranging from oxygen transport, photosynthesis, catalysis and medicine [1–3]. In the past several years, diverse organic scaffolds have been incorporated at the porphyrin periphery and different metal ions in the porphyrin core to modulate ground-state and excited-state characteristics of easily accessible *meso*-tetraarylporphyrins. Some of these π-extended tetrapyrrolic macrocycles have emerged as potential candidates in photodynamic therapy and other materials applications [4–7]. Among the previously synthesized synthetically modified porphyrinoids, β,β’-fused *meso*-tetraphenylporphyrins have gained a considerable importance because of their red-shifted absorption and emission due to the extended π-conjugation. In particular, β,β’-fused quinoxalinoporphyrins displayed a wide range of applications in many fields including molecular electronics [8–10]. Additionally, appropriately functionalized quinoxalinoporphyrin-based photosensitizers are of great interest in the area of dye-sensitized solar cells (DSSC) due to their strong absorption in the visible and near IR regions [11–14]. Similarly, simple quinoxaline-based heterocycles have shown their potential as photosensitizers to induce toxicity in a single cell green algae such as *Chlamydomonas reinhardtii* [15] and also displayed efficacy against *Mycobacterium tuberculosis* and other microbial strains [16–17].

Thorough literature search revealed that the fused heterocycles such as benzo[*a*]pyrano[2,3-*c*]phenazines and benzo[*a*]chromeno[2,3-*c*]phenazines have been prepared as fluorescent materials [18–19]. On the other hand, xanthenes exhibited a number of biological and pharmaceutical profiles such as antimicrobial [20–21], antiviral [22–23], anti-inflammatory [24], anticancer [25], antimalarial agents [26] and are also found to be useful in photodynamic therapy applications [27–29]. In view of above background and also our interest to device convenient protocols for the contruction of periphery-modified porphyrinoids [30–41], we thought to assemble benzo[*f*]chromeno[2,3-*h*]quinoxalinoporphyrins by incorporating porphyrin, quinoxaline and xanthene scaffolds in a single molecular framework using a multicomponent synthetic strategy. The present study discloses an easy and first synthetic approach to build highly π-conjugated copper(II) benzo[*f*]chromeno[2,3-*h*]quinoxalinoporphyrins through a trichloroacetic acid-catalyzed one-pot four-component reaction of 2,3-diamino-5,10,15,20-tetraarylporphyrins, 2-hydroxynaphthalene-1,4-dione, aromatic aldehydes and dimedone in chloroform at 65 °C. The optical properties of the newly prepared porphyrins have been investigated by using UV–vis and emission spectroscopy and the results are presented in this paper.

## Results and Discussion

### Synthesis

The required precursors, copper(II) 2,3-diamino-5,10,15,20-tetraarylporphyrins **1** were synthesized from the corresponding 2-nitro-*meso*-tetraarylporphyrins in two steps by following the literature procedure [42]. The first step involved an amination of copper(II) 2-nitro-*meso*-tetraarylporphyrins by using 4-amino-4*H*-1,2,4-triazole in the presence of NaOH in refluxing ethanol/toluene 1:10 mixture under inert atmosphere to afford 2-amino-3-nitro-*meso*-tetraarylporphyrins which on reduction through sodium borohydride in the presence of 10% Pd/C in CH_2_Cl_2_/MeOH provided the desired porphyrins **1** in good yields as key starting materials for the synthesis of newly designed benzo[*f*]chromeno[2,3-*h*]quinoxalinoporphyrins **3**–**8**. For the optimization of the reaction conditions, a model four-component reaction of copper(II) 2,3-diamino-5,10,15,20-tetra(*p*-tolyl)porphyrin with 2-hydroxynaphthalene-1,4-dione (**2**), benzaldehyde and dimedone was carried out in the presence of 20 mol % *p*-toluenesulfonic acid (PTSA) as an acidic catalyst in chloroform at 65 °C for three hours, which provided copper(II) benzo[*f*]chromeno[2,3-*h*]quinoxalinoporphyrin **3** in 40% yield (Table 1, entry 1). To improve the isolated yield of the desired porphyrin **3**, various experiments were performed by reacting copper(II) 2,3-diamino-5,10,15,20-tetra(*p*-tolyl)porphyrin (**1**) with 2-hydroxynaphthalene-1,4-dione, dimedone and benzaldehyde in the presence of different acidic catalysts such as *p*-toluenesulfonic acid (PTSA), La(OTf)_3_, ʟ-ascorbic acid, *p*-dodecylbenzenesulfonic acid (DBSA), trichloroacetic acid (TCA) and trifluoroacetic acid (TFA) in CHCl_3_ for 3 hours at 65 °C under one-pot operation (Table 1, entries 1–6). Surprisingly, the reaction did not proceed when La(OTf)_3_ and ʟ-ascorbic acid were used as acidic catalysts (Table 1, entries 2 and 3). In contrast, the use of Brønsted acidic catalysts such as DBSA and PTSA afforded porphyrin **3** in only 32% and 40% yield, respectively (Table 1, entries 1 and 4). Interestingly, when trichloroacetic acid (TCA) was used as an acidic catalyst under identical conditions, the output of the reaction was improved giving the desired porphyrin **3** in 65% isolated yield (Table 1, entry 5). However, the reaction in the presence of comparatively strong trifluoroacetic acid (TFA) afforded an inseparable mixture of products under the same conditions (Table 1, entry 6). Hence, trichloroacetic acid was found to be an efficient acidic catalyst for the formation of the targeted porphyrin **3** in good yield. Furthermore, various organic solvents such as 1,2-dichloroethane, toluene, 1,4-dioxane and THF were also screened for the synthesis of porphyrin **3** by using 20 mol % of TCA at 65 °C (Table 1, entries 7–10). When the reaction was carried out in 1,2-dichloroethane and toluene at 65 °C, the desired product **3** was obtained in 58% and 10% yields, respectively (Table 1, entries 7 and 8), whereas the reaction did not proceed by using either 1,4-dioxane or THF as a solvent under otherwise identical reaction conditions (Table 1, entries 9 and 10). Thus, chloroform was found to be the best solvent for the synthesis of porphyrin **3**. Further, the effect of catalyst loading on the rate of reaction was examined by varying the concentration of TCA. The yield of the desired product **3** decreased significantly by lowering the amount of TCA from 20 mol % to 10 mol % (Table 1, entry 11). Whereas no increment in the yield of the desired product **3** was observed when the amount of TCA was increased from 20 mol % to 30 mol % (Table 1, entry 12). Therefore, 20 mol % TCA was found to be sufficient to afford the maximum yield of porphyrin **3**.

**Table 1:** Optimization of the reaction conditions for the synthesis of copper(II) benzo[*f*]chromeno[2,3-*h*]quinoxalinoporphyrin **3**.^a^

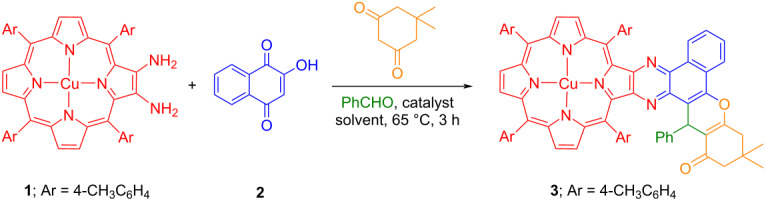				

Entry	Catalyst	Solvent	Time (h)	Yield (%)

1	PTSA (20 mol %)	CHCl_3_	3	40
2	La(OTf)_3_ (20 mol %)	CHCl_3_	3	NR
3	ʟ-ascorbic acid (20 mol %)	CHCl_3_	3	NR
4	DBSA (20 mol %)	CHCl_3_	3	32
**5**	**TCA (20 mol %)**	**CHCl** **_3_**	**3**	**65**
6^b^	TFA (20 mol %)	CHCl_3_	3	0
7	TCA (20 mol %)	ClCH_2_CH_2_Cl	3	58
8	TCA (20 mol %)	toluene	3	10
9	TCA (20 mol %)	1,4-dioxane	3	NR
10	TCA (20 mol %)	THF	3	NR
11	TCA (10 mol %)	CHCl_3_	3	53
12	TCA (30 mol %)	CHCl_3_	3	65
13^c^	TCA (20 mol %)	ClCH_2_CH_2_Cl	3	56
14^d^	TCA (20 mol %)	CHCl_3_	3	34
15^e^	–	CHCl_3_	3	NR
16^e^	–	toluene	6	NR

^a^NR = no reaction; TCA = trichloroacetic acid; PTSA = *p*-toluenesulfonic acid; DBSA = *p*-dodecylbenzenesulfonic acid; TFA = trifluoroacetic acid; ^b^inseparable mixture of products was obtained; ^c^reaction was performed at 80 °C; ^d^reaction was performed at 50 °C; ^e^reaction was carried out at reflux in the absence of TCA.

**Scheme 1:**
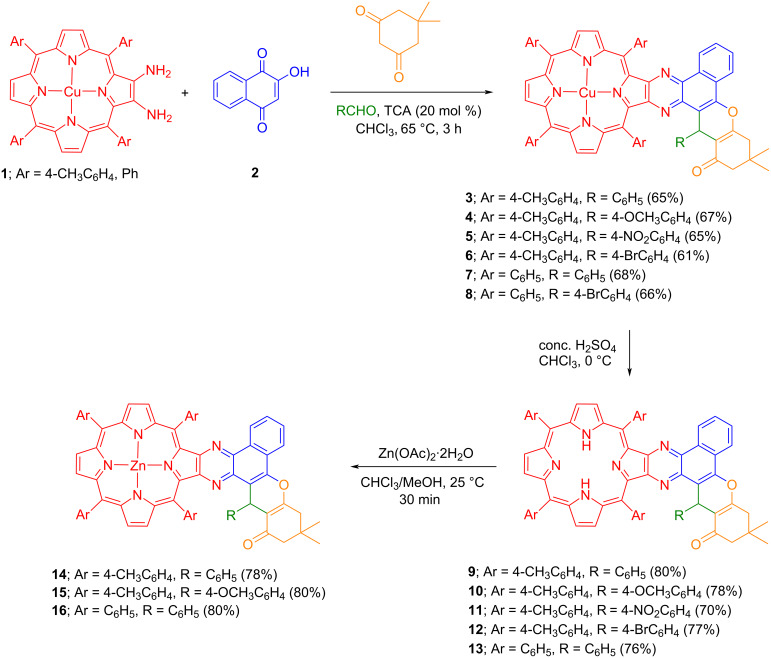
Synthesis of benzo[*f*]chromeno[2,3-*h*]quinoxalinoporphyrins **3**–**16**.

Finally, the effect of temperature was also investigated by performing the experiments at 80 °C in 1,2-dichloroethane and 50 °C in chloroform under the same reaction conditions which produced the desired porphyrin **3** in lower yields (56% and 34%, respectively; Table 1, entries 13 and 14). In contrast, the reaction neither proceeded in chloroform nor in toluene at reflux temperature in the absence of catalyst and always starting material was recovered quantitatively (Table 1, entries 15 and 16). As evident from Table 1, the use of 20 mol % TCA as an acidic catalyst in chloroform at 65 °C was considered to be an optimum condition for the formation of copper(II) benzo[*f*]chromeno[2,3-*h*]quinoxalinoporphyrin **3** in appreciable yield. Further, a new series of copper(II) benzo[*f*]chromeno[2,3-*h*]quinoxalinoporphyrins **3**–**8** were constructed in good isolated yields by using the optimized reaction conditions (Scheme 1).

For a comparative study of absorption and emission properties, the copper complexes of benzo[*f*]chromeno[2,3-*h*]quinoxalinoporphyrins **3***–***7** were converted to the corresponding free-base porphyrinoids **9***–***13** through a standard demetallation process using conc. H_2_SO_4_ in CHCl_3_ under cooling conditions (Scheme 1). On complexation with zinc by using Zn(OAc)_2_ in CHCl_3_/MeOH, free-base porphyrins **9**, **10** and **13** afforded zinc(II) benzo[*f*]chromeno[2,3-*h*]quinoxalinoporphyrins **14***–***16** in good yields (Scheme 1).

**Figure 1:**
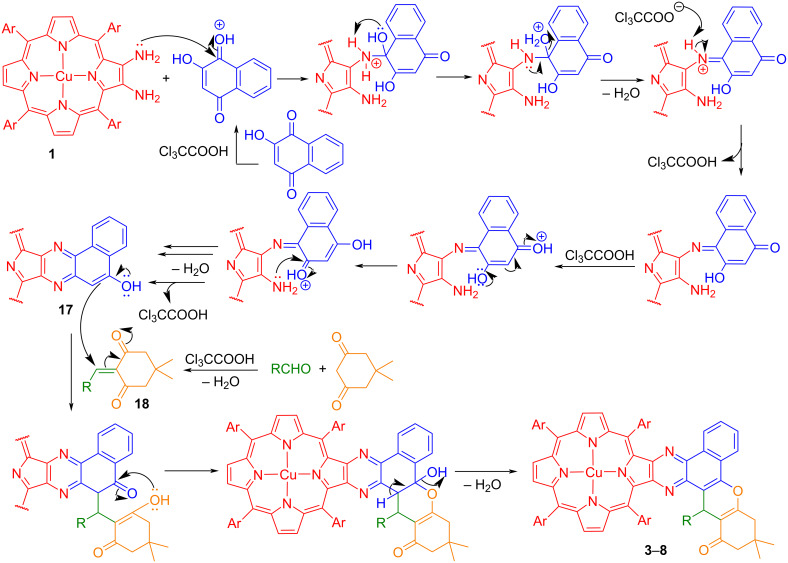
Plausible mechanism for the formation of copper(II) benzo[*f*]chromeno[2,3-*h*]quinoxalinoporphyrins.

The proposed mechanistic pathway for the formation of copper(II) benzo[*f*]chromeno[2,3-*h*]quinoxalinoporphyrins **3***–***8** under one-pot operation is presented in Figure 1. At the beginning of the reaction, copper(II) 2,3-diamino-5,10,15,20-tetraarylporphyrins **1** react with 2-hydroxynaphthalene-1,4-dione (**2**) in the presence of trichloroacetic acid to form an imine intermediate which on intramolecular cyclization affords a key benzo[*f*]quinoxalinoporphyrin intermediate **17**. Further, a condensation of intermediate **17** with 2-arylidene-5,5-dimethylcyclohexane-1,3-dione **18** (formed in situ through an Aldol condensation of aldehydes with dimedone), to generate copper(II) benzo[*f*]chromeno[2,3-*h*]dihydroquinoxalinoporphyrins which on dehydration produce the desired copper(II) benzo[*f*]chromeno[2,3-*h*]quinoxalinoporphyrins **3**–**8** in 61–68% yields.

**Scheme 2:**
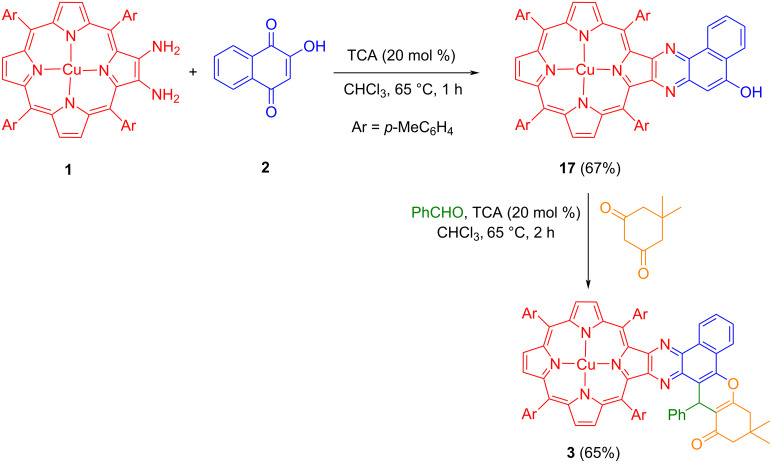
Sequential synthesis of copper(II) benzo[*f*]chromeno[2,3-*h*]quinoxalinoporphyrin **3**.

To authenticate the proposed reaction pathway, a control experiment was carried out by reacting copper(II) 2,3-diamino-5,10,15,20-tetrakis(4-methylphenyl)porphyrin (**1**) with 2-hydroxynaphthalene-1,4-dione (**2**) in CHCl_3_ containing 20 mol % TCA at 65 °C as presented in Scheme 2. After workup and chromatographic purification, the isolated product was characterized based on spectral data analysis as copper(II) benzo[*f*]quinoxalinoporphyrin intermediate **17**. Further, porphyrin **17** reacted with benzaldehyde and dimedone in chloroform containing 20 mol% trichloroacetic acid at 65 °C to afford copper(II) benzo[*f*]chromeno[2,3-*h*]quinoxalinoporphyrin **3** in 65% yield (Scheme 2). The successful isolation of intermediate **17** and its conversion to copper(II) benzo[*f*]chromeno[2,3-*h*]quinoxalinoporphyrin **3** as shown in Scheme 2 clearly support the proposed mechanism for the formation of the desired copper(II) porphyrins **3**–**8**.

Finally, the structures of all newly synthesized benzo[*f*]chromeno[2,3-*h*]quinoxalinoporphyrins **3**–**16** and benzo[*f*]quinoxalinoporphyrin **17** were assigned on the basis of IR, ^1^H and ^13^C NMR, and HRMS data analysis.

### Photophysical characteristics

**Figure 2:**
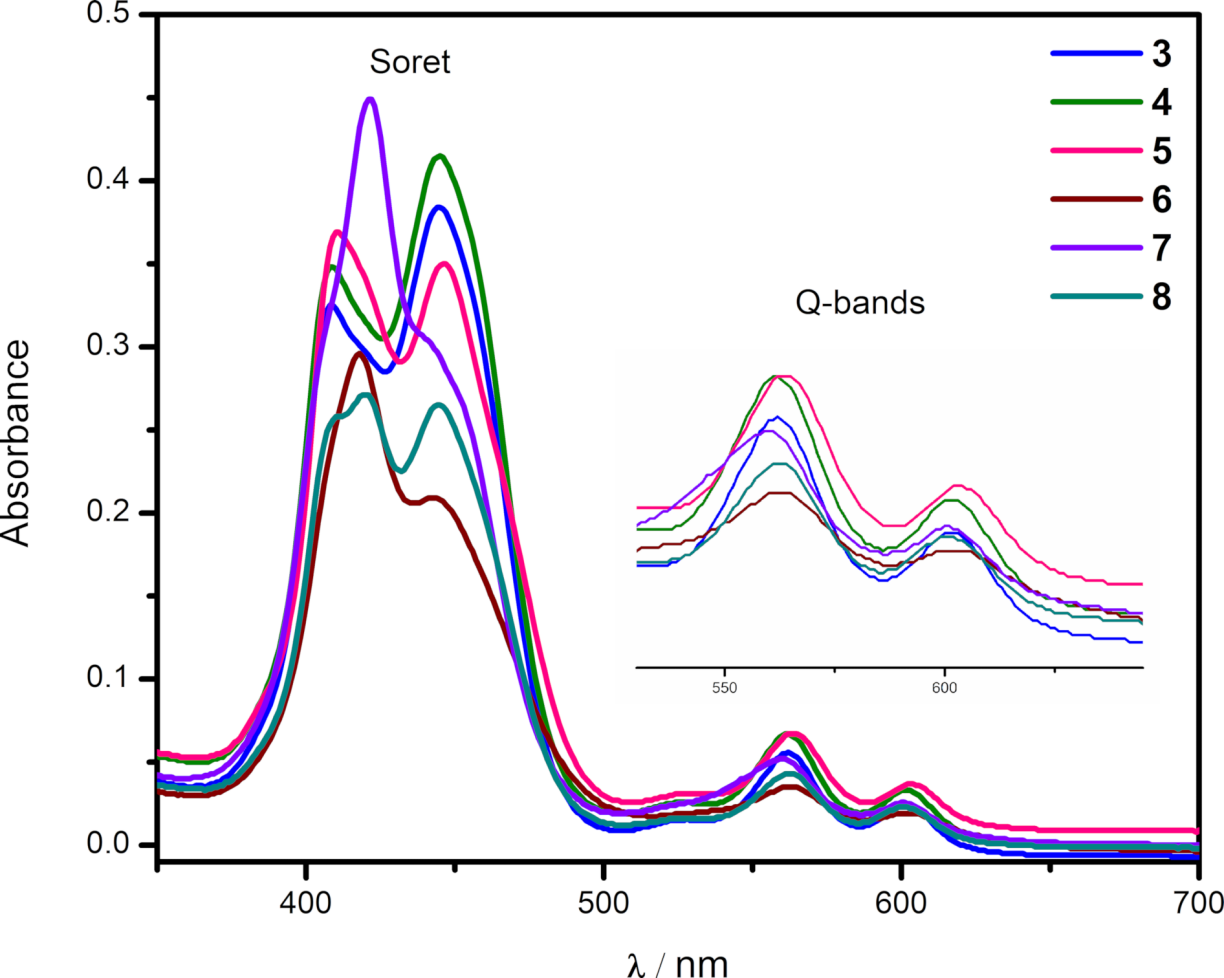
Electronic absorption spectra of copper(II) benzo[*f*]chromeno[2,3-*h*]quinoxalinoporphyrins **3**–**8** in CHCl_3_ (1.5 × 10^−6^ M) at 298 K. (Inset shows Q-bands).

**Figure 3:**
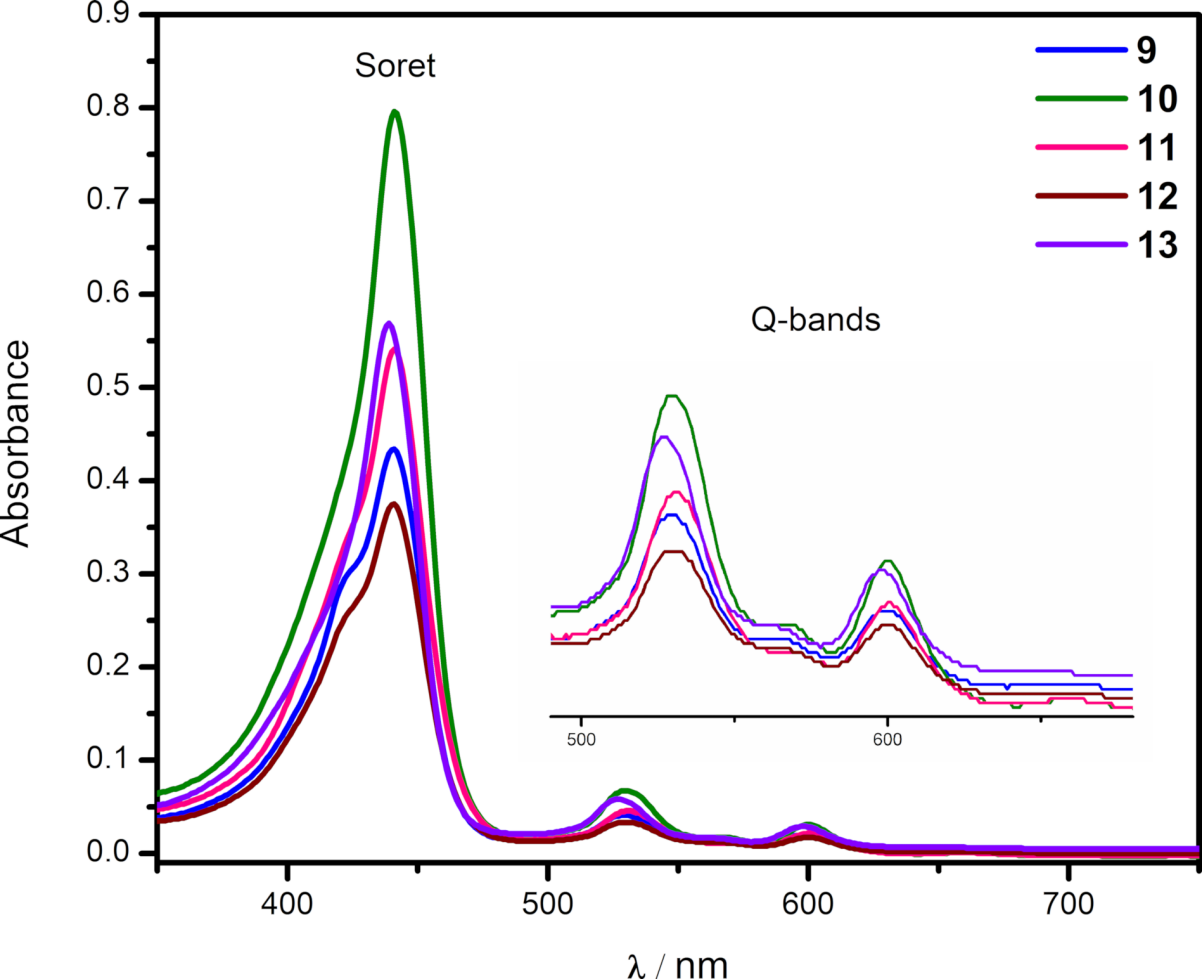
Electronic absorption spectra of free-base benzo[*f*]chromeno[2,3-*h*]quinoxalinoporphyrins **9**–**13** in CHCl_3_ (1.5 × 10^−6^ M) at 298 K. (Inset shows Q-bands).

**Figure 4:**
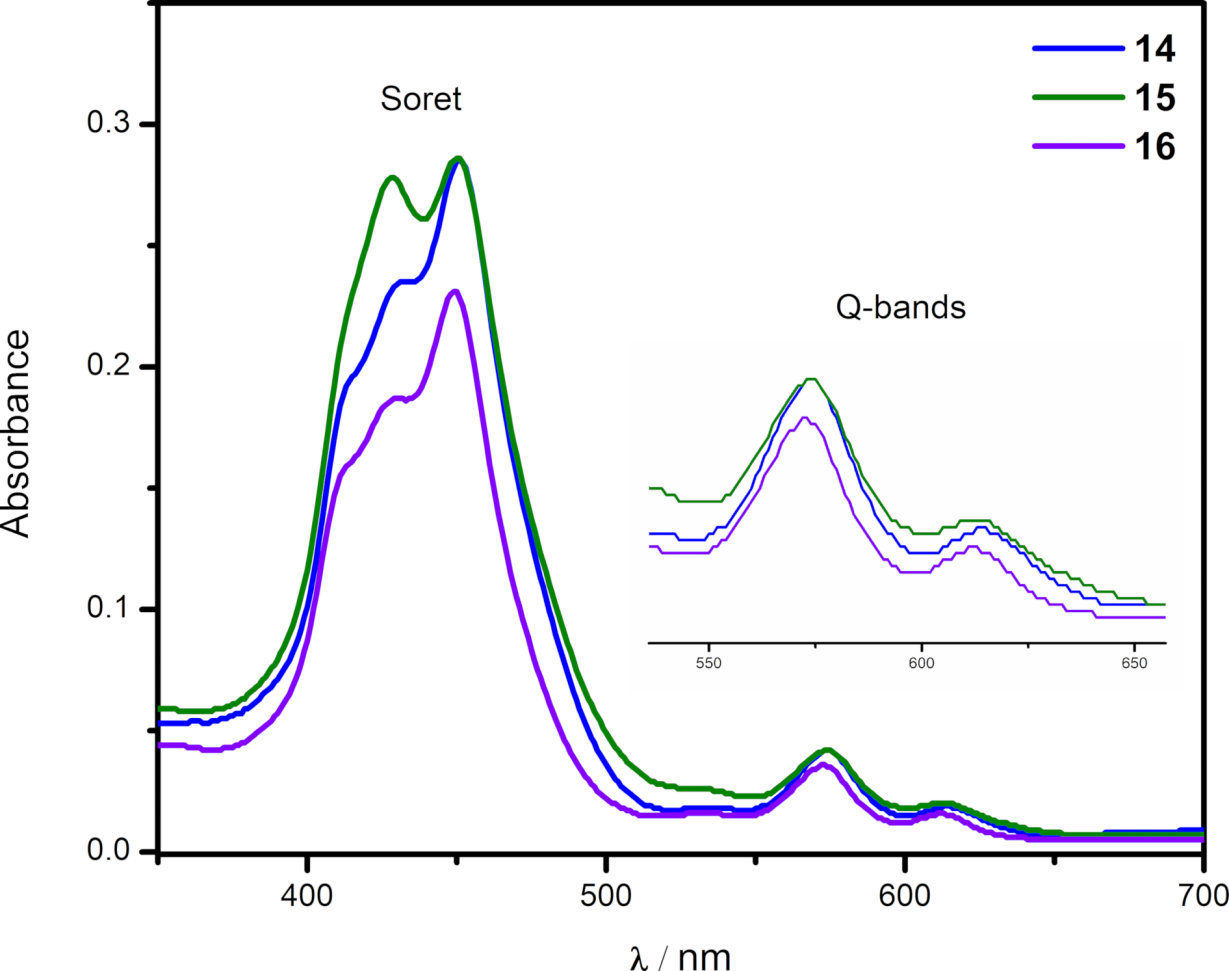
Electronic absorption spectra of zinc(II) benzo[*f*]chromeno[2,3-*h*]quinoxalinoporphyrins **14**–**16** in CHCl_3_ (1.5 × 10^−6^ M) at 298 K. (Inset shows Q-bands).

The UV–vis spectra of the newly synthesized benzo[*f*]chromeno[2,3-*h*]quinoxalinoporphyrins **3**–**16** (Figures 2–4) were recorded in chloroform at 25 °C. Interestingly, the absorption spectra of copper(II) benzo[*f*]chromeno[2,3-*h*]quinoxalinoporphyrins **3**–**8** are significantly broadened and feature split Soret bands between 408–445 nm probably due to the loss in *D*_4_*_h_* symmetry after the fusion of a large benzo[*f*]chromeno[2,3-*h*]quinoxaline moiety across the porphyrinic β-positions [43], and two Q-bands at ≈562 and 603 nm (Figure 2). Further, the electronic absorpion spectra of free-base benzo[*f*]chromeno[2,3-*h*]quinoxalinoporphyrins **9**–**13** exhibited Soret bands between 439–442 nm and four Q-bands between 526 and 642 nm (Figure 3). In contrast, UV–visible spectra of zinc(II) benzo[*f*]chromeno[2,3-*h*]quinoxalinoporphyrins **14**–**16** showed broadened Soret bands with slight splitting between 445–450 nm and two Q-bands at ≈566 and 606 nm (Figure 4).

All the newly prepared copper(II), free-base and zinc(II) benzo[*f*]chromeno[2,3-*h*]quinoxalinoporphyrins displayed a significant red-shift in their Soret and Q-bands by ≈20–30 nm as compared to their corresponding *meso*-tertakis(4-methylphenyl)porphyrins (Cu-TMPP, Soret band at 416 nm; TMPP, Soret band at 419 nm; Zn-TMPP, Soret band at 425 nm) due to the extended π-conjugation after the fusion of the benzo[*f*]chromeno[2,3-*h*]quinoxaline moiety at the β-pyrrolic positions of the porphyrin macrocycle.

**Figure 5:**
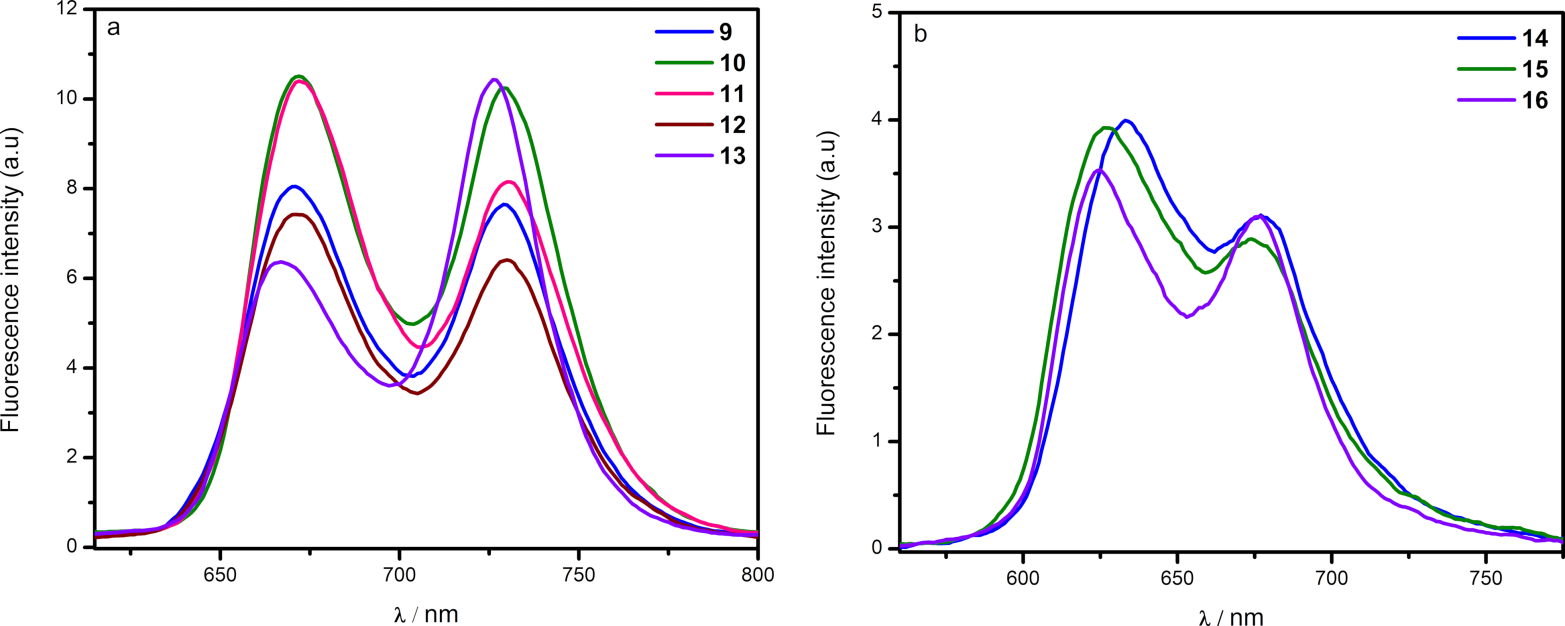
(a) Emission spectra of free-base benzo[*f*]chromeno[2,3-*h*]quinoxalinoporphyrins **9**–**13** and (b) emission spectra of zinc(II) benzo[*f*]chromeno[2,3-*h*]quinoxalinoporphyrins **14**–**16** in CHCl_3_ (1.5 × 10^−6^ M) at 298 K (λ_Ex_ = 420 nm).

In the fluorescence spectra, free-base benzo[*f*]chromeno[2,3-*h*]quinoxalinoporphyrins **9**–**13** showed emission bands at ≈675 nm and 730 nm (Figure 5a). These newly synthesized free-base porphyrins displayed significant red-shifts in their emission spectra in comparison to *meso*-tetrakis(4-methylphenyl)porphyrin (TMPP; emission bands at ≈652 and 717 nm). Similarly, fluorescence spectra of zinc(II) benzo[*f*]chromeno[2,3-*h*]quinoxalinoporphyrins **14**, **15** and **16** (Figure 5b) showed two emission bands between 623 and 678 nm with a red-shift of 21–25 nm in comparison to zinc(II) *meso*-tetrakis(4-methylphenyl)porphyrin (Zn-TMPP; emission bands at 602 and 653 nm). However, no emission was observed in the case of copper(II) porphyrins due to the paramagnetic nature of copper(II) ions [44].

## Conclusion

In summary, we have successfully synthesized a new series of copper(II) benzo[*f*]chromeno[2,3-*h*]quinoxalinoporphyrin analogues in good yields following the one-pot synthetic strategy applying the reaction of copper(II) 2,3-diamino-*meso*-tetraarylporphyrins with 2-hydroxynaphthalene-1,4-dione, aromatic aldehydes and dimedone in the presence of 20 mol % trichloroacetic acid in chloroform at 65 °C. Interestingly, a sequential approach for constructing copper(II) benzo[*f*]chromeno[2,3-*h*]quinoxalinoporphyrin **3** was also followed by capturing a key intermediate, copper(II) benzo[*f*]quinoxalinoporphyrin **17** for the mechanistic studies. On photophysical evaluation, the newly synthesized porphyrins displayed significant red-shifted absorption and emission as compared to simple *meso*-tetraarylporphyrins due to the extended π-electronic conjugation. Hence, the present study is potentially useful for the development of highly conjugated π-electron rich porphyrinoids with improved light harvesting properties.

## Experimental

### Materials and instrumentation methods

All reagents and solvents used in this study were purchased from Sigma-Aldrich (Merck) and were used as received unless otherwise stated. The column chromatographic purifications of all products were carried out using either activated neutral aluminium oxide (Brokmann grade I-II, Merck). The melting points of all newly prepared products were determined on a Büchi M-560 melting point apparatus. ^1^H NMR (400 MHz) and ^13^C NMR (100 MHz) spectra were recorded in CDCl_3_ on a Jeol ECX-400P (400 MHz) NMR spectrometer. Chemical shifts are reported in δ scale in parts per million (ppm) relative to CDCl_3_ (δ = 7.26 ppm for ^1^H NMR and δ = 77.00 ppm for ^13^C NMR). The coupling constants are expressed as (*J*) and are reported in hertz (Hz). Infrared (IR) spectra of the synthesized compounds were recorded in film or KBr on Perkin Elmer IR spectrometer and absorption maxima (υ_max_) are given in cm^−1^. UV–vis absorption and fluorescence spectra were recorded on an Analytik Jena’s Specord 250 UV–vis spectrophotometer and a Varian Cary Eclipse fluorescence spectrophotometer, respectively. The mass spectra were recorded on an Agilent G 6530 AA LC-HRMS QTOF system in positive mode. Spectroscopic grade chloroform was used to measure UV–visible and emission spectra of the samples. Thin-layer chromatography (TLC) was performed on silica gel 60 F_254_ (pre-coated aluminium) sheets from Merck.

### General procedure for the synthesis of copper(II) benzo[*f*]chromeno[2,3-*h*]quinoxalinoporphyrins **3**–**8**

To a solution of copper(II) 2,3-diamino-*meso*-tetraarylporphyrin (**1**; 0.131 mmol) in chloroform (20 mL), 2-hydroxynaphthalene-1,4-dione (**2**; 0.157 mmol) and trichloroacetic acid (0.026 mmol) were added and the reaction mixture was stirred at reflux temperature for 30 minutes. Then, the aromatic aldehyde (0.157 mmol) and dimedone (0.157 mmol) were added and the reaction mixture was refluxed for additional two and a half hours. The progress of the reaction was monitored by TLC. After completion of the reaction, the product was extracted by using chloroform (3 × 50 mL). The organic layers were combined and washed with water (3 × 50 mL), dried over anhydrous sodium sulfate and evaporated under reduced pressure. The crude product was purified on a neutral alumina column by using 20% chloroform in hexane as eluent to afford porphyrins **3**–**8** in 61–68% yields.

### General procedure for the synthesis of free-base benzo[*f*]chromeno[2,3-*h*]quinoxalinoporphyrins **9**–**13**

To a solution of copper(II) benzo[*f*]chromeno[2,3-*h*]quinoxalinoporphyrin **3**–**7** (0.090 mmol) in chloroform (20 mL), conc. H_2_SO_4_ (1.6 mL) was added and the reaction mixture was stirred at 0 °C for 7 min. After completion of the reaction, the reaction mixture was quenched with water and neutralized with saturated sodium bicarbonate solution. The resulting mixture was extracted with chloroform (50 mL). The organic layer was washed with water (3 × 50 mL), dried over anhydrous sodium sulfate and evaporated under reduced pressure. The crude product was purified on a neutral alumina column by using 40% chloroform in hexane as eluent to afford porphyrins **9**–**13** in 70–80% yields.

### General procedure for the synthesis of zinc(II) benzo[*f*]chromeno[2,3-*h*]quinoxalinoporphyrins **14**–**16**

To a solution of free-base benzo[*f*]chromeno[2,3-*h*]quinoxalinoporphyrins **9**, **10** and **13** (0.038 mmol) in chloroform (10 mL), a solution of Zn(OAc)_2_·2H_2_O (0.136 mmol) in methanol (2 mL) was added and reaction mixture was stirred at 25 °C for thirty minutes. The progress of the reaction was monitored by TLC. After completion of the reaction, the reaction mixture was diluted with chloroform (50 mL). The resulting solution was washed with water (3 × 50 mL), the organic layer was dried over anhydrous sodium sulfate and evaporated under reduced pressure. The crude product was purified on activated neutral alumina column by using 80% chloroform in hexane as eluent to afford porphyrins **14**–**16** in 78–80% yields.

### Synthesis of copper(II) benzo[*f*]quinoxalinoporphyrin **17**

To a solution of copper(II) 2,3-diamino-*meso*-tetraarylporphyrin **1** (0.131 mmol) in chloroform (15 mL), 2-hydroxynaphthalene-1,4-dione (**2**; 0.157 mmol) and trichloroacetic acid (0.026 mmol) were added and the reaction mixture was stirred at reflux temperature for 2 hours and the progress of the reaction was monitored by TLC. After completion of the reaction, the crude product was extracted by using chloroform (3 × 50 mL). The organic layer was washed with water (3 × 50 mL), dried over anhydrous sodium sulfate and evaporated under reduced pressure. The crude product obtained was purified on a neutral alumina column by using 20% chloroform in hexane as eluent to afford porphyrin **17** in 67% yield.

## Supporting Information

File 1 Characterization data, ^1^H and ^13^C NMR spectra of newly prepared porphyrin products.
