# Supplementary material for: Unravelling a trichloroacetic acid-catalyzed cascade access to benzo[f]chromeno[2,3-h]quinoxalinoporphyrins
Source: Beilstein J Org Chem. 2023 Aug 11;19:1216–24. doi: 10.3762/bjoc.19.89 (PMC10428578; doi:10.3762/bjoc.19.89)
Supplement: File 1 — Characterization data, 1H and 13C NMR spectra of newly prepared porphyrin products. [file Beilstein_J_Org_Chem-19-1216-s001.pdf]

BEILSTEIN JOURNAL OF ORGANIC CHEMISTRY

Supporting Information

for

Unravelling a trichloroacetic acid-catalyzed cascade access to benzo[*f*]chromeno[2,3-*h*]quinoxalinoporphyrins

Chandra Sekhar Tekuri, Pargat Singh and Mahendra Nath

Beilstein J. Org. Chem. **2023,** *19,* 1216–1224. doi:10.3762/bjoc.19.89

Characterization data, ^{1}H and ^{13}C NMR spectra of newly prepared porphyrin products

License and Terms: This is a supporting information file under the terms of the Creative Commons Attribution License (https://creativecommons.org/licenses/by/4.0). Please note that the reuse, redistribution and reproduction in particular requires that the author(s) and source are credited and that individual graphics may be subject to special legal provisions.
The license is subject to the *Beilstein Journal of Organic Chemistry* terms and conditions: (https://www.beilstein-journals.org/bjoc/terms)

CONTENTS PAGE

Characterization data of copper(II) benzo[f]chromeno[2,3-h]quinoxalino-porphyrins 3–8 S2

Characterization data of free-base benzo[f]chromeno[2,3-h]quinoxalino-porphyrins 9–13 S3

Characterization data of zinc(II) benzo[f]chromeno[2,3-h]quinoxalino-porphyrins 14–16 S5

Characterization data of copper(II) benzo[f]quinoxalinoporphyrin 17 S7

^{1}H and ^{13}C NMR spectra of free-base and zinc(II) porphyrins 9–16 S8

Characterization data of copper(II) benzo[*f*]chromeno[2,3-*h*]quinoxalino-porphyrins 3–8

Copper(II) benzo[*f*]chromeno[2,3-*h*]quinoxalinoporphyrin 3, Ar = 4-$CH_3C_6H_4$, **R** = C_6H_5

Purple solid; yield: 65%; mp >300°C; IR ($CHCl_3$) ν_{max}: 755, 796, 1003, 1039, 1179, 1210, 1344, 1450, 1507, 1597, 1659, 2872, 2957, 3021 cm^{-1}; UV λ_{max} ($\varepsilon \times 10^{-4}$, M^{-1} cm^{-1}): 408 (21.6), 445 (25.6), 562 (3.7), 604 (1.6) nm; ESI-HRMS (m/z) calcd for $C_{73}H_{55}N_6O_2Cu$: 1110.3677 $[M + H]^+$; found 1110.3607.

Copper(II) benzo[*f*]chromeno[2,3-*h*]quinoxalinoporphyrin 4, Ar = 4-$CH_3C_6H_4$, **R =** 4-$OCH_3C_6H_4$

Purple solid; yield: 67%; mp >300°C; IR ($CHCl_3$) ν_{max}: 758, 797, 1004, 1039, 1112, 1179, 1211, 1244, 1345, 1456, 1509, 1606, 1660, 2857, 2923, 3020 cm^{-1}; UV λ_{max} ($\varepsilon \times 10^{-4}$, M^{-1} cm^{-1}): 409 (23.2), 445 (27.6), 562 (4.4), 602 (2.2) nm; ESI-HRMS (m/z) calcd for $C_{74}H_{57}N_6O_3Cu$: 1140.3783 $[M + H]^+$; found 1140.3784.

Copper(II) benzo[*f*]chromeno[2,3-*h*]quinoxalinoporphyrin 5, Ar = 4-$CH_3C_6H_4$, **R =** 4-$NO_2C_6H_4$

Purple solid; yield: 65%; mp >300°C; IR ($CHCl_3$) ν_{max}: 763, 798, 1004, 1181, 1213, 1345, 1458, 1518, 1661, 1744, 2323, 2365, 2854, 2923, 3021 cm^{-1}; UV λ_{max} ($\varepsilon \times 10^{-4}$, M^{-1} cm^{-1}): 410 (24.6), 447 (23.3), 564 (4.4), 603 (2.4) nm; ESI-HRMS (m/z) calcd for $C_{73}H_{54}N_7O_4Cu$: 1155.3528 $[M + H]^+$; found 1155.3525.

Copper(II) benzo[*f*]chromeno[2,3-*h*]quinoxalinoporphyrin 6, Ar = 4-$CH_3C_6H_4$, **R** = 4-BrC_6H_4

Purple solid; yield: 61%; mp >300°C; IR ($CHCl_3$) ν_{max}: 753, 796, 1004, 1178, 1211, 1259, 1344, 1455, 1511, 1656, 2858, 2921, 2958, 3018 cm^{-1}; UV λ_{max} ($\varepsilon \times 10^{-4}$, M^{-1} cm^{-1}): 418 (19.3), 444 (13.3), 564 (2.0), 602 (0.6) nm; ESI-HRMS (m/z) calcd for $C_{73}H_{54}BrN_6O_2Cu$: 1188.2782 $[M + H]^+$; found 1188.2777.

Copper(II) benzo[*f*]chromeno[2,3-*h*]quinoxalinoporphyrin 7, Ar = C_6H_5, **R =** C_6H_5

Purple solid; yield: 68%; mp >300°C; IR ($CHCl_3$) ν_{max}: 700, 750, 796, 1003, 1071, 1212, 1344, 1448, 1514, 1596, 1656, 2957, 3020 cm^{-1}; UV λ_{max} ($\varepsilon \times 10^{-4}$, M^{-1} cm^{-1}): 422 (29.9), 443 (19.9) 561 (3.4), 601 (1.7) nm; ESI-HRMS (m/z) calcd for $C_{69}H_{47}N_6O_2Cu$: 1054.3051 $[M + H]^+$; found 1054.3062.

Copper(II) benzo[*f*]chromeno[2,3-*h*]quinoxalinoporphyrin 8, Ar = C_6H_5, **R =** 4-BrC_6H_4

Purple solid; yield: 66%; mp >300°C; IR ($CHCl_3$) ν_{max}: 759, 797, 1005, 1179, 1211, 1344, 1453, 1510, 1659, 2852, 2921, 3021 cm^{-1}; UV λ_{max} ($\varepsilon \times 10^{-4}$, M^{-1} cm^{-1}): 421 (18.0), 445 (17.6), 562 (2.8), 600 (1.5) nm; ESI-HRMS (m/z) calcd for $C_{69}H_{46}N_6O_2BrCu$: 1132.2156 $[M + H]^+$; found 1132.2150.

Characterization data of free-base benzo[*f*]chromeno[2,3-*h*]quinoxalino-porphyrins 9–13

Free-base benzo[*f*]chromeno[2,3-*h*]quinoxalinoporphyrin 9, Ar = 4-$CH_3C_6H_4$, **R** = C_6H_5

Purple solid; yield: 80%; mp >300°C; IR ($CHCl_3$) ν_{max}: 669, 751, 795, 970, 1031, 1154, 1209, 1301, 1347, 1460, 1509, 1598, 1656, 2850, 2917, 3018, 3346 cm^{-1}; ^1H NMR (400 MHz, $CDCl_3$) δ_H: -2.56 (s, 2H, internal NH), 1.17(s, 3H, CH_3), 1.57 (s, 3H, CH_3), 2.47(dd, *J* = 16.02 Hz, 2H, CH_2), 2.71-2.72 (m, 6H, CH_3), 2.82 (s, 2H, CH_2), 2.86 (s, 3H, CH_3), 2.94 (s, 3H, CH_3), 5.79 (s, 1H, CH), 6.82 (t, *J* = 7.63 Hz, 1H, ArH), 6.93 (t, *J* = 7.63 Hz, 2H, ArH), 7.16 (d, *J* = 7.63 Hz, 2H, ArH), 7.54-7.84 (m, 11H, *meso*-ArH, ArH), 8.00-8.22 (m, 7H, *meso*-ArH), 8.40 (d, *J* = 7.63 Hz, 1H, ArH), 8.63 (d, *J* = 7.63 Hz, 1H, ArH), 8.75 (s, 2H, *β*-pyrrolic H), 8.91-8.98 (m, 4H, *β*-pyrrolic H) ppm; ^{13}C NMR (100 MHz, $CDCl_3$) δ_C: 14.13, 21.53, 21.74, 21.90, 27.25, 32.16, 32.37, 41.41, 51.28, 115.48, 117.08, 117.98, 118.17, 121.23, 121.47, 125.73, 125.76, 127.41, 127.49, 127.53, 127.75, 127.88, 128.10, 128.14, 128.36, 128.58, 128.76, 131.56, 133.90, 134.12, 134.18, 134.40, 134.48, 134.57, 137.24, 137.28, 137.45, 137.48, 138.10, 138.20, 138.36, 138.61, 138.75, 138.87, 139.05, 139.48, 139.63, 140.33, 144.79, 145.51, 146.71, 149.98, 151.76, 154.89, 155.11, 164.12, 196.25 ppm; UV λ_{max} ($\varepsilon \times 10^{-4}$, M^{-1} cm^{-1}): 441 (28.9), 529 (2.7), 568 (0.3), 600 (1.3), 642 (0.1) nm; λ_{Em} ($CHCl_3$; λ_{Ex} 420 nm): 668, 730 nm; ESI-HRMS (m/z) calcd for $C_{73}H_{57}N_6O_2$: 1049.4538 $[M + H]^+$; found 1049.4558.

Free-base benzo[*f*]chromeno[2,3-*h*]quinoxalinoporphyrin 10, Ar = 4-$CH_3C_6H_4$, **R** = 4-$OCH_3C_6H_4$

Purple solid; yield: 78%; mp >300°C; IR ($CHCl_3$) ν_{max}: 755 796, 972, 1039, 1170, 1210, 1302, 1372, 1463, 1509, 1606, 1658, 2592, 2679, 2858, 2922, 3018, 3348 cm^{-1}; ^1H NMR (400 MHz, $CDCl_3$) δ_H: -2.36 (s, 2H, internal NH), 1.33 (s, 3H, CH_3), 1.41 (s, 3H, CH_3), 2.63 (dd, *J* = 16.02, Hz, 2H, CH_2), 2.85-2.86 (m, 6H, CH_3), 2.97 (s, 2H, CH_2), 3.05 (s, 3H, CH_3), 3.08 (s, 3H, CH_3), 3.64 (s, 3H, OCH_3), 5.91 (s, 1H, CH), 6.65 (d, *J* = 9.16, Hz, 2H, ArH), 7.28 (s, 1H, ArH), 7.69-7.76 (m, 4H, *meso*-ArH, ArH), 7.83-7.96 (m, 6H, *meso*-ArH), 8.07-8.10 (m, 1H, *meso*-ArH), 8.20-8.39 (m, 8H, *meso*-ArH), 8.55 (d, *J* = 7.63 Hz, 1H, ArH), 8.78 (d, *J* = 8.39, Hz, 1H, ArH), 8.92 (s, 2H, *β*-pyrrolic H), 9.09-9.15 (m, 4H, *β*-pyrrolic H) ppm; ^{13}C NMR (100 MHz, $CDCl_3$)

δ_C: 14.13, 21.51, 21.72, 21.93, 27.25, 31.44, 32.37, 41.38, 51.29, 54.83, 112.77, 115.58, 117.11, 118.16, 118.20, 121.21, 121.24, 121.48, 125.75, 125.77, 127.50, 127.52, 127.74, 127.90, 128.12, 128.16, 128.59, 128.74, 129.39, 131.52, 133.93, 134.13, 134.45, 134.56, 135.24, 137.23, 137.32, 137.44, 137.47, 138.11, 138.22, 138.42, 138.67, 138.75, 138.86, 139.00, 139.50, 139.62, 140.36, 144.80, 145.58, 146.52, 149.97, 151.76, 154.90, 155.13, 157.45, 163.85, 196.31 ppm; UV λ_{max} ($\varepsilon \times 10^{-4}$, M^{-1} cm^{-1}): 441 (53.0), 529 (4.4), 570 (0.5), 600 (2.0), 640 (0.1) nm; λ_{Em} ($CHCl_3$; λ_{Ex} 440 nm): 672, 728 nm; ESI-HRMS (m/z) calcd for $C_{74}H_{59}N_6O_3$: 1079.4643 [M + H]$^+$; found 1079.4649.

Free-base benzo[*f*]chromeno[2,3-*h*]quinoxalinoporphyrin 11, Ar = 4-$CH_3C_6H_4$, **R =** 4-$NO_2C_6H_4$

Purple solid; yield: 70%; mp >300°C; IR ($CHCl_3$) ν_{max}: 722, 761, 797, 971, 1040, 1162, 1211, 1304, 1345, 1464, 1518, 1599, 1661, 2854, 2923, 3023, 3348 cm^{-1}; ^1H NMR (400 MHz, $CDCl_3$) δ_H: -2.55 (s, 2H, internal NH), 1.14 (s, 3H, CH_3), 1.56 (s, 3H, CH_3), 2.48 (dd, *J* = 16.02, 2H, CH_2), 2.71-2.72 (m, 6H, CH_3), 2.85 (s, 2H, CH_2), 2.88 (s, 3H, CH_3), 2.93 (s, 3H, CH_3), 5.87 (s, 1H, CH), 7.29-7.31 (m, 2H, ArH), 7.57-7.92 (m, 14H, *meso*-ArH, ArH), 8.07-8.22 (m, 6H, *meso*-ArH), 8.42 (d, *J* = 7.63 Hz, 1H, ArH), 8.64 (d, *J* = 7.63 Hz, 1H, ArH), 8.74 (s, 2H, *β*-pyrrolic H), 8.89-8.95 (m, 4H, *β*-pyrrolic H) ppm; UV λ_{max} ($\varepsilon \times 10^{-4}$, M^{-1} cm^{-1}): 441 (21.8), 530 (3.8), 569 (0.4), 600 (2.6), 638 (0.1) nm; λ_{Em} ($CHCl_3$; λ_{Ex} 430 nm): 672, 730 nm; ESI-HRMS (m/z) calcd for $C_{73}H_{56}N_7O_4$: 1094.4388 [M + H]$^+$; found 1094.4395.

Free-base benzo[*f*]chromeno[2,3-*h*]quinoxalinoporphyrin 12, Ar = 4-$CH_3C_6H_4$, **R** = 4-BrC_6H_4

Purple solid; yield: 77%; mp >300°C; IR ($CHCl_3$) ν_{max}:755, 796, 971, 1041, 1156, 1209, 1302, 1368, 1472, 1510, 1600, 1659, 2860, 2920, 2956, 3020, 3345 cm^{-1}; ^1H NMR (400 MHz, $CDCl_3$) δ_H: -2.54 (s, 2H, internal NH), 1.16 (s, 3H, CH_3), 1.27 (s, 3H, CH_3), 2.48 (dd, *J* = 16.02, 2H, CH_2), 2.72 (s, 6H, CH_3), 2.83 (s, 2H, CH_2), 2.89 (s, 3H, CH_3), 2.94 (s, 3H, CH_3), 5.76 (s, 1H, CH), 7.02-7.07 (m, 4H, ArH), 7.58-7.88 (m, 10H, *meso*-ArH, ArH), 8.04-8.24 (m, 7H, *meso*-ArH), 8.35-8.41 (m, 2H, ArH), 8.64 (d, *J* = 8.39 Hz, 1H, ArH), 8.75-8.77 (m, 2H, *β*-pyrrolic H), 8.90-8.97 (m, 4H, *β*-pyrrolic H) ppm; ^{13}C NMR (100 MHz, $CDCl_3$) δ_C: 21.53. 21.74, 21.89, 27.21, 29.79, 31.97, 32.38, 41.37, 51.21, 114.95, 117.10, 117.33, 118.05, 119.65, 121.27, 121.56, 125.58, 125.81, 127.51, 127.54, 127.76, 127.94, 128.09, 128.21, 128.60, 128.85, 130.30, 130.46, 131.64, 133.95, 134.18, 134.54, 137.26, 137.29, 137.48, 137.51, 138.13, 138.26, 138.43, 138.46, 138.70, 138.83, 139.03, 139.48, 139.63, 140.34, 143.87, 144.61, 145.27, 146.60, 150.07, 151.68, 154.94, 155.19, 164.16, 196.22 ppm; UV λ_{max} ($\varepsilon \times 10^{-4}$, M^{-1}

cm^{-1}): 441 (25.0), 529 (2.2), 568 (0.3), 600 (1.1), 639 (0.1) nm; λ_{Em} ($CHCl_3$; λ_{Ex} 440 nm): 671, 730 nm; ESI-HRMS (m/z) calcd for $C_{73}H_{56}N_6O_2Br$: 1127.3643 [M + H]$^+$; found 1127.3647.

Free-base benzo[*f*]chromeno[2,3-*h*]quinoxalinoporphyrin 13, Ar = C_6H_5, **R =** C_6H_5 Purple solid; yield: 76%; mp >300°C IR ($CHCl_3$) ν_{max}: 750, 798, 968, 1158, 1207, 1347, 1440, 1596, 1657, 2882, 2960, 3022, 3058, 3344 cm^{-1}; ^{1}H NMR (400 MHz, $CDCl_3$) δ_H: -2.56 (s, 2H, internal NH), 1.19 (s, 3H, CH_3), 1.28 (s, 3H, CH_3), 2.51 (dd, J_1 = 16.78 Hz and J_2 = 16.02 Hz, 2H, CH_2), 2.83 (s, 2H, CH_2), 5.77 (s, 1H, CH), 6.84-6.87 (m, 1H, *meso*-ArH), 6.96 (t, J = 7.63 Hz, 2H, ArH), 7.19 (d, J = 8.39 Hz, 2H, ArH), 7.74-7.94 (m, 11H, *meso*-ArH), 8.01-8.05 (m, 2H, ArH), 8.10-8.22 (m, 4H, *meso*-ArH), 8.29-8.34 (m, 4H, *meso*-ArH), 8.40 (d, J = 7.63 Hz, 1H, ArH), 8.47-8.49 (m, 1H, ArH), 8.58 (d, J = 8.39 Hz, 1H, ArH), 8.75 (s, 2H, β-pyrrolic H), 8.92-8.96 (m, 4H, β-pyrrolic H) ppm; ^{13}C NMR (100 MHz, $CDCl_3$) δ_C: 27.20, 29.90, 31.76, 32.41, 41.40, 51.30, 115.56, 117.13, 117.92, 118.24, 121.20, 121.28, 121.53, 125.71, 125.82, 125.87, 126.79, 127.12, 127.24, 127.39, 127.43, 127.56, 127.84, 127.97, 128.25, 128.65, 128.86, 129.29, 131.43, 134.04, 134.22, 134.45, 134.57, 134.92, 135.01, 137.24, 138.04, 138.09, 138.71, 139.52, 140.10, 141.76, 141.89, 142.52, 144.59, 144.78, 145.52, 147.03, 150.08, 151.85, 154.84, 155.03, 164.67, 196.76 ppm; UV λ_{max} ($\varepsilon \times 10^{-4}$, M^{-1} cm^{-1}): 439 (37.9), 527 (3.8), 567 (0.5), 597 (1.9), 637 (0.1) nm; λ_{Em} ($CHCl_3$; λ_{Ex} 435 nm): 668, 726 nm; ESI-HRMS (m/z) calcd for $C_{69}H_{49}N_6O_2$: 993.3912 [M + H]$^+$; found 993.3905.

Characterization data of zinc(II) benzo[*f*]chromeno[2,3-*h*]quinoxalino-porphyrins 14–16

Zinc(II) benzo[*f*]chromeno[2,3-*h*]quinoxalinoporphyrin 14, Ar = 4-$CH_3C_6H_4$, **R** = C_6H_5 Purple solid; yield: 78%; mp >300°C; IR ($CHCl_3$) ν_{max}: 758, 796, 1000, 1039, 1175, 1212, 1303, 1340, 1371, 1406, 1450, 1505, 1605, 1653, 2859, 2922, 3021 cm^{-1}; ^{1}H NMR (400 MHz, $CDCl_3$) δ_H: 1.18 (s, 6H, CH_3), 2.47(dd, J = 15.26 Hz, 2H, CH_2), 2.72 (s, 6H, CH_3), 2.83 (s, 2H, CH_2), 2.86 (s, 3H, CH_3), 2.94 (s, 3H, CH_3), 5.86 (s, 1H, CH), 6.84 (t, J = 6.87 Hz, 1H, ArH), 6.94 (d, J = 7.63 Hz, 2H, ArH), 7.16 (d, J = 7.63 Hz, 2H, ArH), 7.53-7.58 (m, 4H, *meso*-ArH), 7.64 (t, J = 6.10 Hz, 2H, ArH), 7.73-7.79 (m, 4H, *meso*-ArH), 7.99-8.09 (m, 4H, *meso*-ArH), 8.16 (t, J = 6.87 Hz, 3H, *meso*-ArH), 8.26 (d, J = 6.87 Hz, 1H, *meso*-ArH), 8.37 (d, J = 7.63 Hz, 1H, ArH), 8.63 (d, J = 7.63 Hz, 1H, ArH), 8.81 (d, J = 4.58 Hz, 1H, β-pyrrolic H), 8.88 (s, 2H, β-pyrrolic H), 8.92-8.97 (m, 3H, β-pyrrolic H) ppm; ^{13}C NMR (100 MHz, $CDCl_3$) δ_C: 21.53, 21.75, 21.94, 27.25, 29.83, 32.15, 32.37, 41.40, 51.28, 115.55, 117.56, 117.92, 118.79, 121.25, 122.99, 123.28, 125.78, 125.96, 127.33, 127.42, 127.62, 127.66, 127.83, 127.94,

128.39, 128.90, 131.33, 131.46, 131.54, 132.02, 132.17, 133.48, 133.76, 134.17, 134.29, 134.47, 136.89, 137.19, 137.21, 137.67, 138.24, 139.00, 139.11, 139.61, 139.63, 140.31, 140.61, 141.04, 144.75, 146.94, 147.81, 149.15, 149.20, 149.79, 149.86, 149.99, 152.36, 153.07, 164.20, 196.26 ppm; UV λ_{max} ($\varepsilon \times 10^{-4}$, M^{-1} cm^{-1}): 450 (19.0), 574 (2.8), 615 (1.2) nm; λ_{Em} ($CHCl_3$; λ_{Ex} 430 nm): 632, 675 nm; ESI-HRMS (m/z) calcd for $C_{73}H_{55}N_6O_2Zn$: 1111.3672 $[M + H]^+$; found 1111.3694.

Zinc(II) benzo[*f*]chromeno[2,3-*h*]quinoxalinoporphyrin 15, Ar = 4-$CH_3C_6H_4$, **R** = 4-$OCH_3C_6H_4$

Purple solid; yield: 80%; mp >300°C; IR ($CHCl_3$) ν_{max}: 759, 796, 1001, 1039, 1175, 1213, 1340, 1371, 1454, 1509, 1607, 1651, 2858, 2923, 3019 cm^{-1}; ^1H NMR (400 MHz, $CDCl_3$) δ_H: 1.16 (s, 3H, CH_3), 1.25 (s, 3H, CH_3), 2.45 (dd, *J* = 15.26, 2H, CH_2), 2.71 (s, 6H, CH_3), 2.81 (s, 2H, CH_2), 2.86 (s, 3H, CH_3), 2.93 (s, 3H, CH_3), 3.46 (s, 3H, OCH_3), 5.77 (s, 1H, CH), 6.46 (d, *J* = 9.16 Hz, 2H, ArH), 7.07 (d, *J* = 8.39 Hz, 2H, ArH), 7.52-7.63 (m, 6H, *meso*-ArH, ArH), 7.73-7.82 (m, 3H, *meso*-ArH), 7.96-8.19 (m, 8H, *meso*-ArH), 8.28 (d, *J* = 8.39 Hz, 1H, ArH), 8.38 (d, *J* = 7.63 Hz, 1H, ArH), 8.65 (d, *J* = 8.39 Hz, 1H, ArH), 8.81 (d, *J* = 4.58 Hz, 1H, *β*-pyrrolic H), 8.86 (s, 2H, *β*-pyrrolic H), 8.91-8.95 (m, 3H, *β*-pyrrolic H) ppm; ^{13}C NMR (100 MHz, $CDCl_3$) δ_C: 21.53, 21.73, 21.91, 27.24, 29.84, 31.39, 32.36, 41.36, 51.24, 54.82, 112.74, 115.63, 117.53, 118.11, 118.71, 121.15, 122.95, 123.22, 125.73, 125.88, 127.32, 127.49, 127.59, 127.63, 127.84, 127.88, 128.80, 129.37, 131.32, 131.34, 131.43, 131.52, 131.97, 132.15, 133.45, 133.78, 134.17, 134.23, 134.31, 134.56, 136.83, 137.15, 137.18, 137.23, 137.64, 138.25, 139.06, 139.08, 139.62, 140.30, 140.60, 141.00, 146.68, 147.78, 149.11, 149.17, 149.77, 149.82, 149.95, 152.34, 153.03, 157.40, 163.92, 196.33 ppm; UV λ_{max} ($\varepsilon \times 10^{-4}$, M^{-1} cm^{-1}): 428 (18.5), 451 (19.0), 574 (2.8), 618 (1.2) nm; λ_{Em} ($CHCl_3$; λ_{Ex} 435 nm): 625, 677 nm; ESI-HRMS (m/z) calcd for $C_{74}H_{57}N_6O_3Zn$: 1141.3778 $[M + H]^+$; found 1141.3775.

Zinc(II) benzo[*f*]chromeno[2,3-*h*]quinoxalinoporphyrin 16, Ar = C_6H_5, **R =** C_6H_5

Purple solid; yield: 80%; mp >300°C; IR ($CHCl_3$) ν_{max}: 698, 750, 795, 831, 999, 1069, 1173, 1211, 1338, 1444, 1483, 1597, 1652, 2852, 2920, 2954, 3020, 3056 cm^{-1}; ^1H NMR (400 MHz, $CDCl_3$) δ_H: 1.24 (s, 3H, methyl H), 1.19 (s, 3H, methyl H), 2.48 (dd, *J* = 16.78, 2H, CH_2), 2.84 (s, 2H, CH_2), 5.81(s, 1H, CH), 6.85 (t, *J* = 7.63 Hz, 1H, ArH), 6.96 (t, *J* = 7.63 Hz, 2H, ArH), 7.19 (d, *J* = 7.63 Hz, 2H, ArH), 7.73-7.84 (m, 10H, *meso*-ArH), 7.91-7.96 (m, 2H, *meso*-ArH), 8.01 (t, *J* = 7.63 Hz, 1H, ArH), 8.08-8.22 (m, 5H, *meso*-ArH), 8.26-8.30 (m, 3H, *meso*-ArH), 8.39 (d, *J* = 7.63 Hz, 2H, ArH), 8.61 (d, *J* = 7.63 Hz, 1H, ArH), 8.86-8.88 (m, 4H, *β*-pyrrolic H), 8.92-8.95 (m, 2H, *β*-pyrrolic H) ppm; ^{13}C NMR (100 MHz, $CDCl_3$) δ_C: 27.18, 31.75, 32.40,

41.38, 51.26, 115.61, 117.60, 117.84, 118.83, 121.21, 122.99, 123.26, 125.75, 125.89, 125.99, 126.58, 126.97, 127.01, 127.12, 127.28, 127.47, 127.55, 127.60, 128.28, 129.00, 131.32, 131.43, 131.54, 131.63, 132.06, 132.13, 132.24, 133.59, 133.88, 134.09, 134.21, 134.31, 134.47, 137.63, 139.06, 140.55, 141.02, 141.86, 142.54, 143.28, 144.54, 147.19, 147.87, 149.11, 149.77, 149.89, 152.37, 152.82, 164.70, 196.77 ppm; UV λ_{max} ($\varepsilon \times 10^{-4}$, M^{-1} cm^{-1}): 449 (15.4), 572 (2.4), 613 (1.0) nm; λ_{Em} ($CHCl_3$; λ_{Ex} 440 nm): 623, 678 nm; ESI-HRMS (m/z) calcd for $C_{69}H_{47}N_6O_2Zn$: 1055.3046 $[M + H]^+$; found 1055.3046.

Characterization data of copper(II) benzo[*f*]quinoxalinoporphyrin 17, Ar = 4-$CH_3C_6H_4$ Purple solid; yield: 67%; mp >300°C; IR ($CHCl_3$) ν_{max}: 796, 1004, 1183, 1222, 1346, 1453, 1511, 1604, 1689, 2856, 2923, 3023 cm^{-1}; UV λ_{max} ($\varepsilon \times 10^{-4}$, M^{-1} cm^{-1}): 441 (13.8), 559 (3.0), 600 (1.4) nm; ESI-HRMS (m/z) calcd for $C_{58}H_{41}CuN_6O$: 900.2632 $[M + H]^+$; found 900.2635.

^{1}H and ^{13}C NMR spectra of free-base and zinc(II) porphyrins 9–16

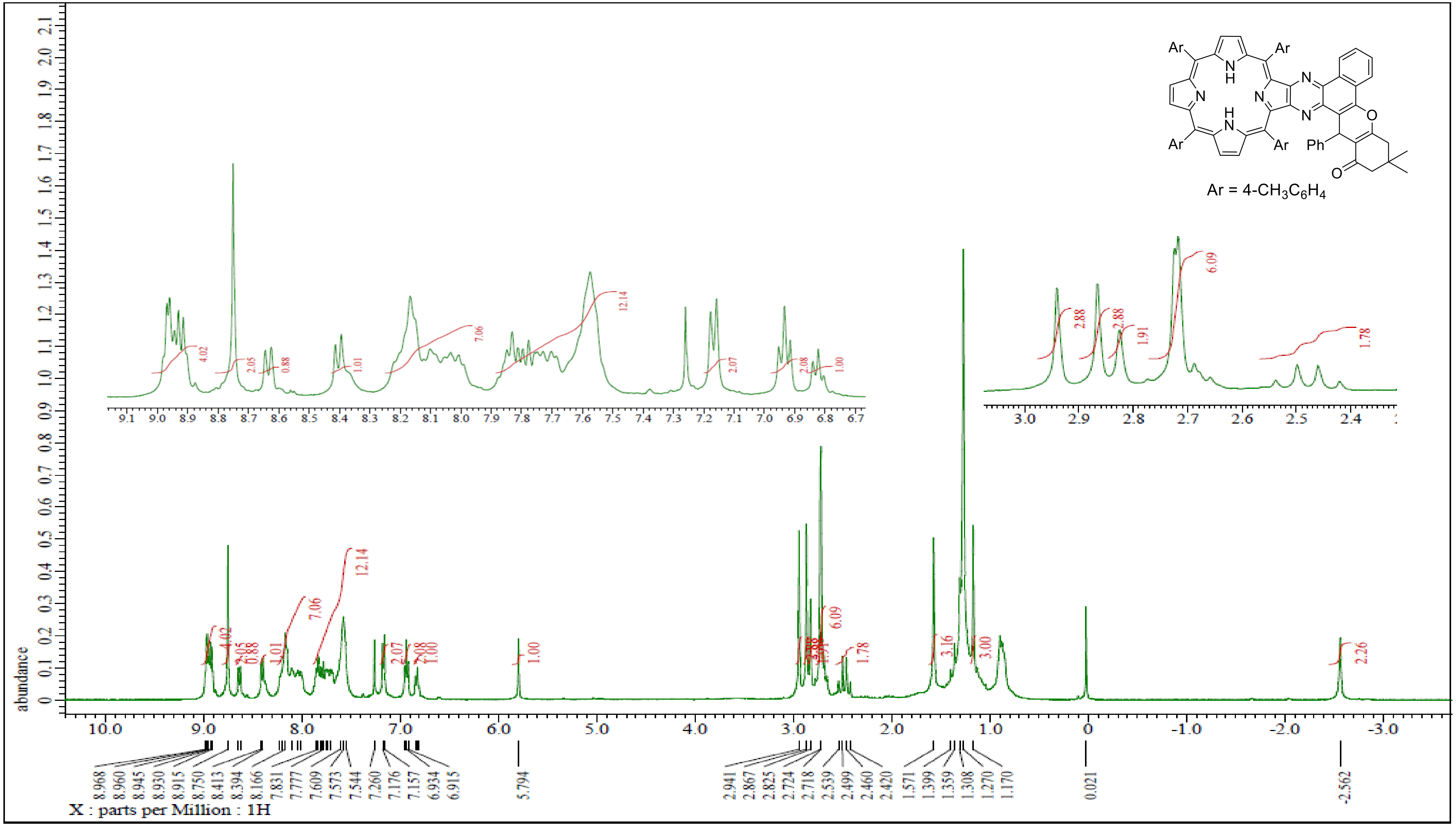

Figure 1. ^{1}H NMR spectrum of porphyrin **9** in $CDCl_3$.

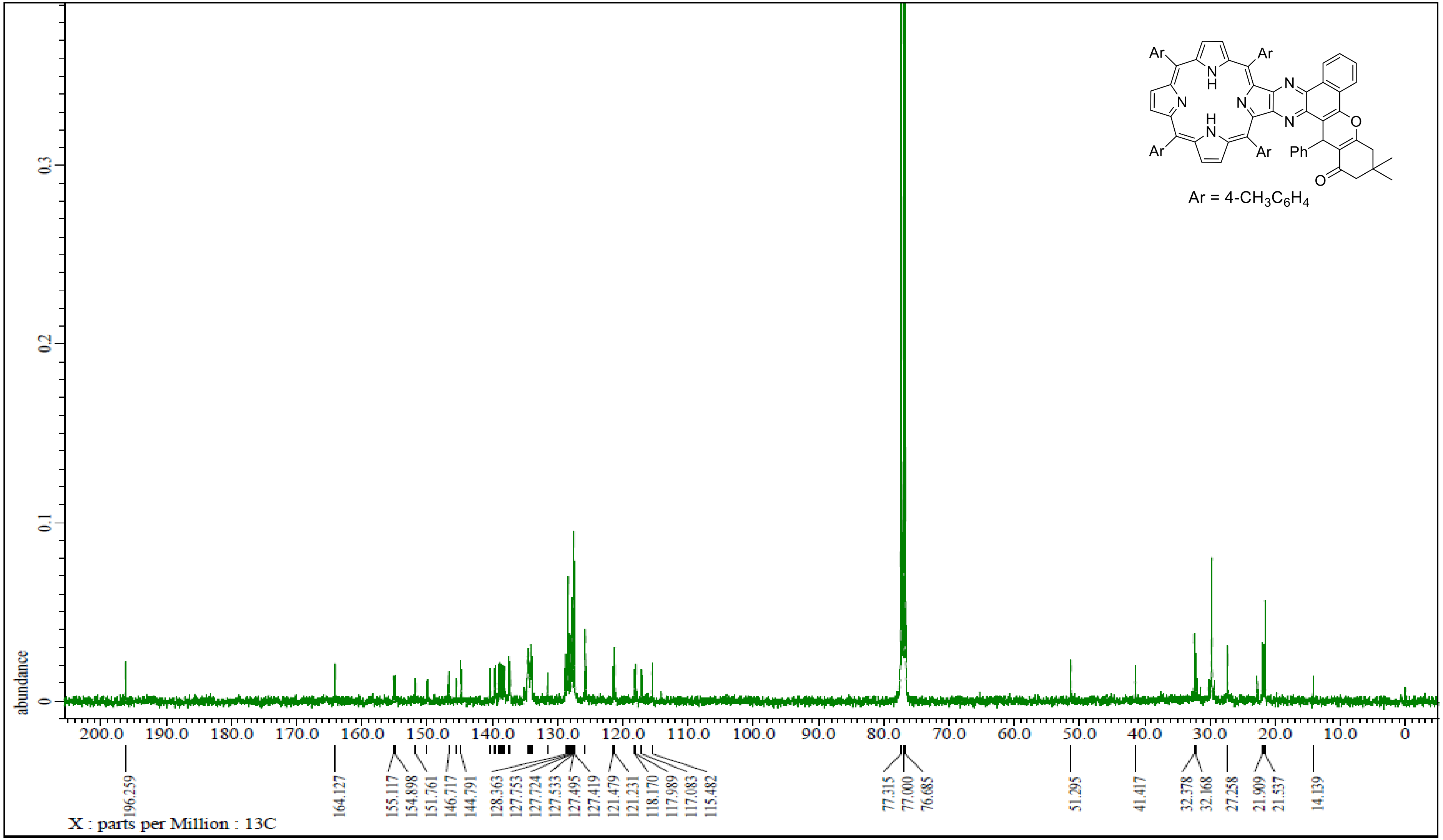

Figure 2. ^{13}C NMR spectrum of porphyrin **9** in $CDCl_3$.

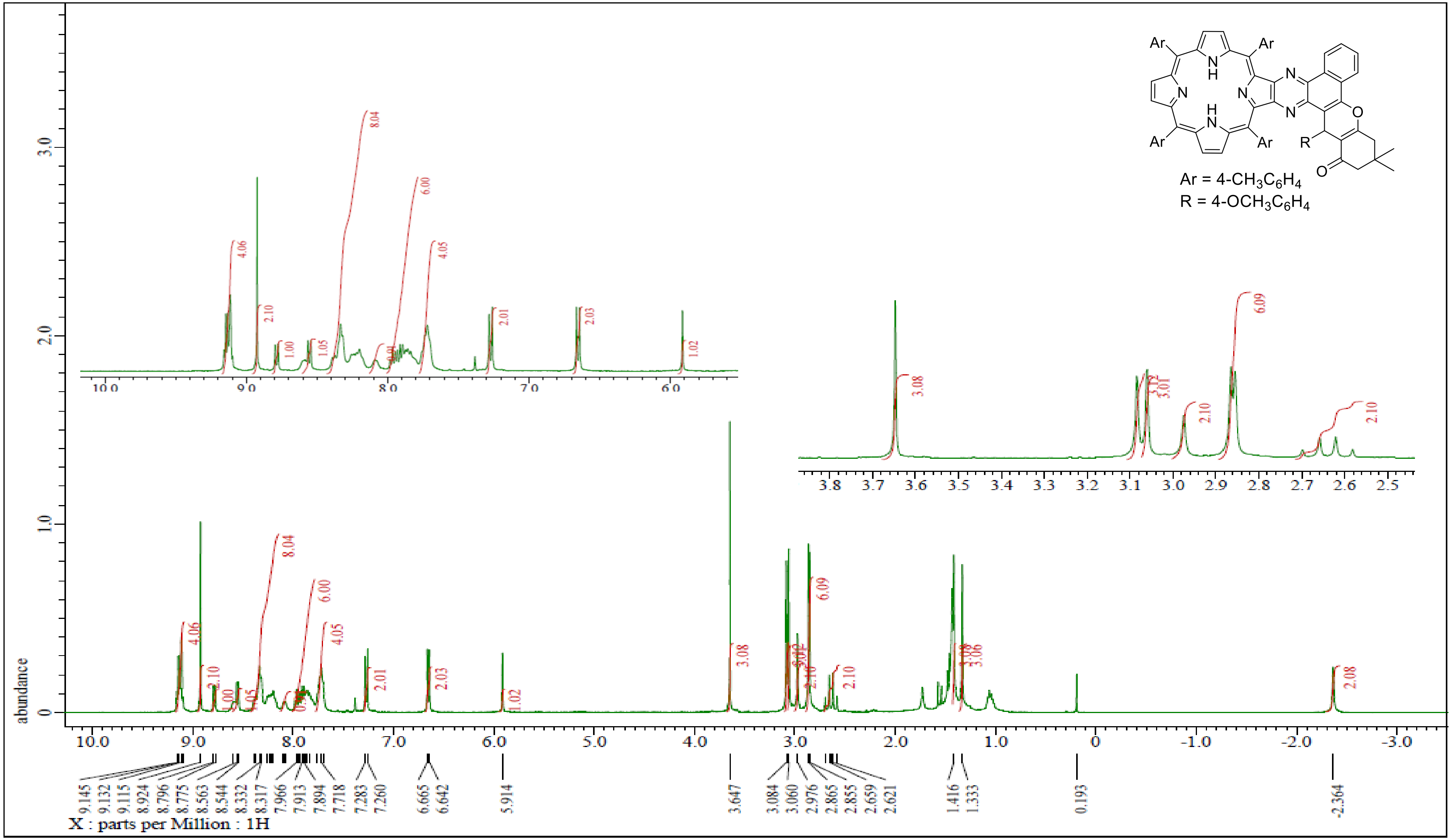

Figure 3. ^{1}H NMR spectrum of porphyrin **10** in $CDCl_3$.

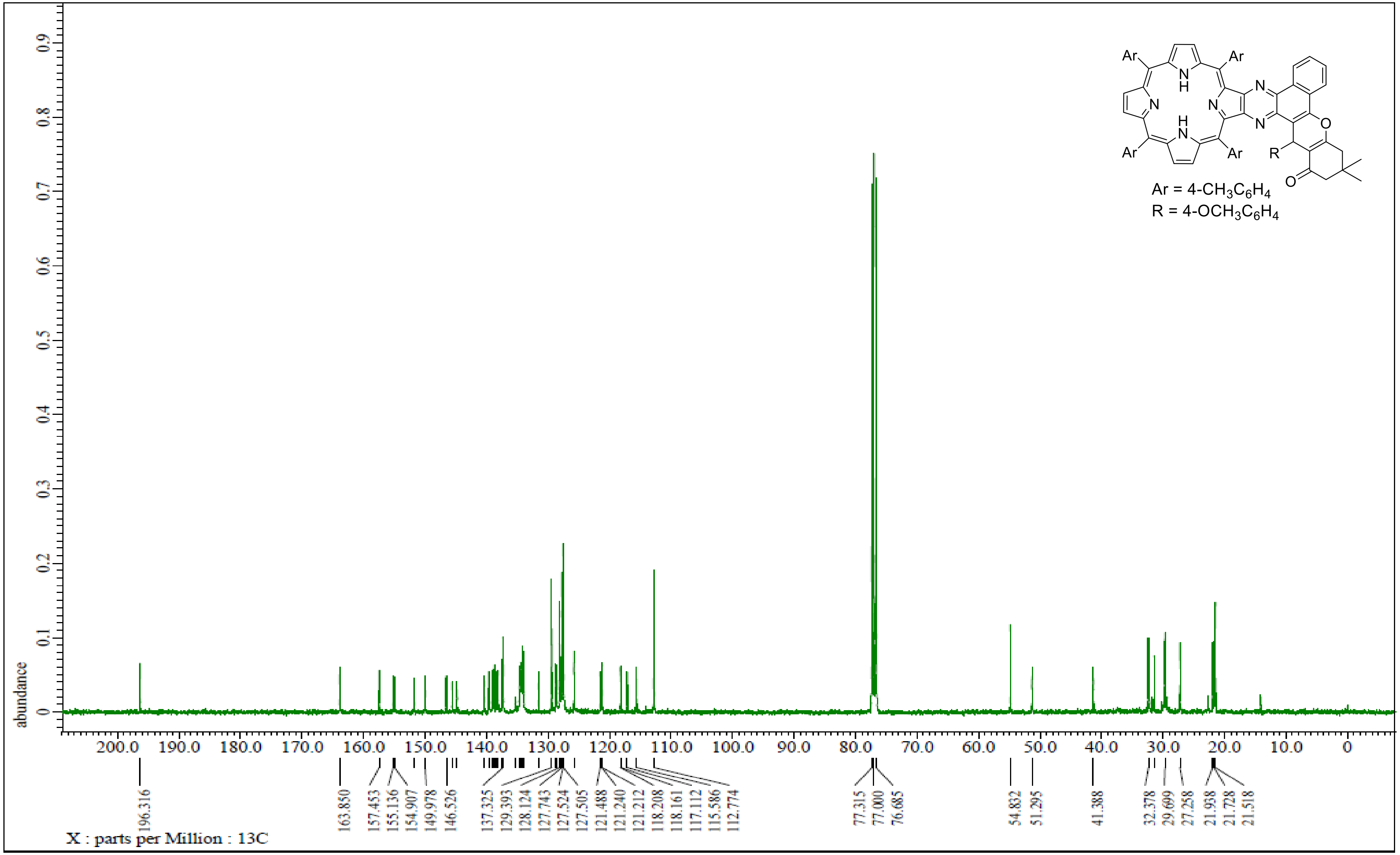

Figure 4. ^{13}C NMR spectrum of porphyrin **10** in $CDCl_3$.

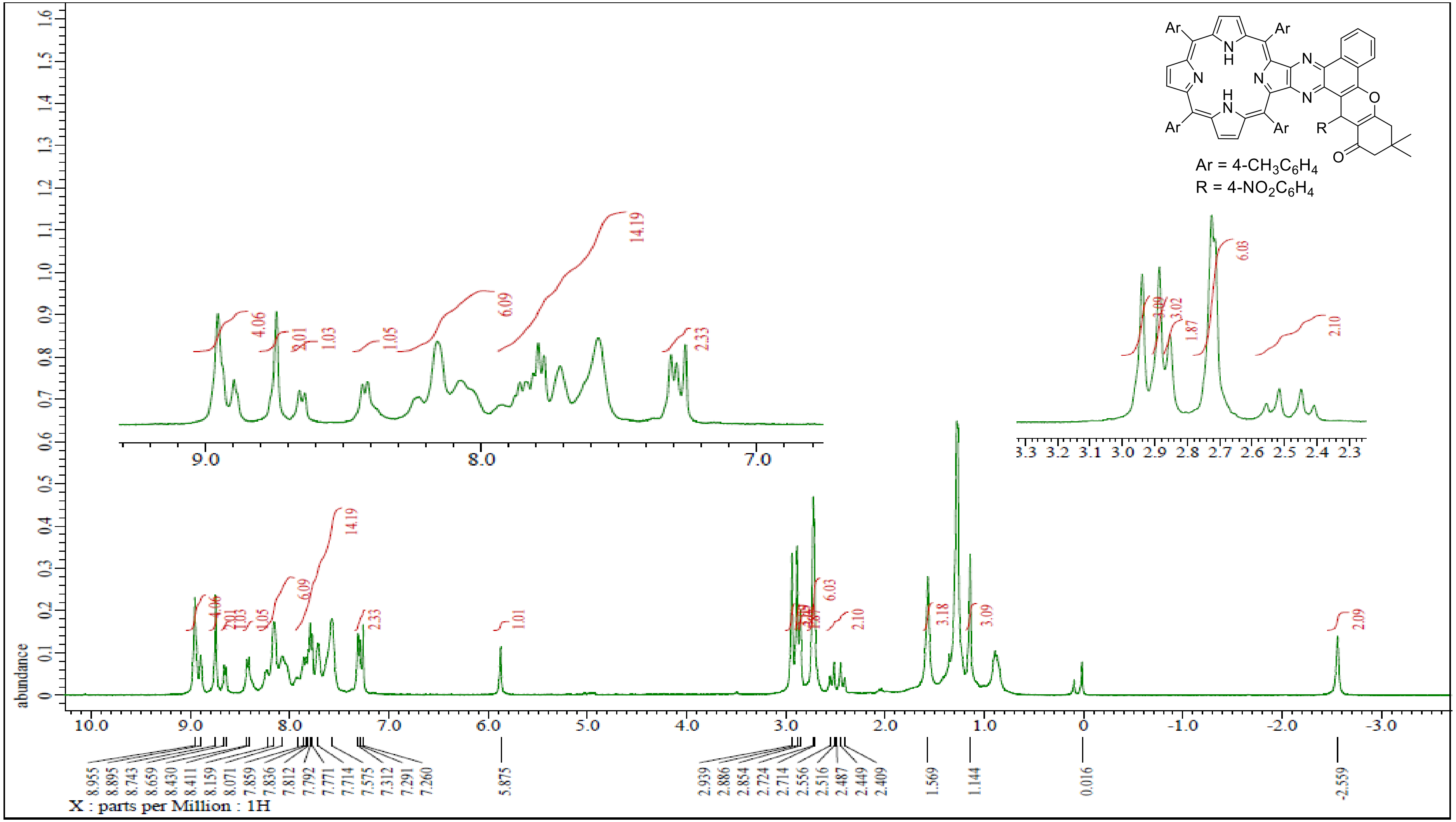

Figure 5. ^{1}H NMR spectrum of porphyrin **11** in $CDCl_3$.

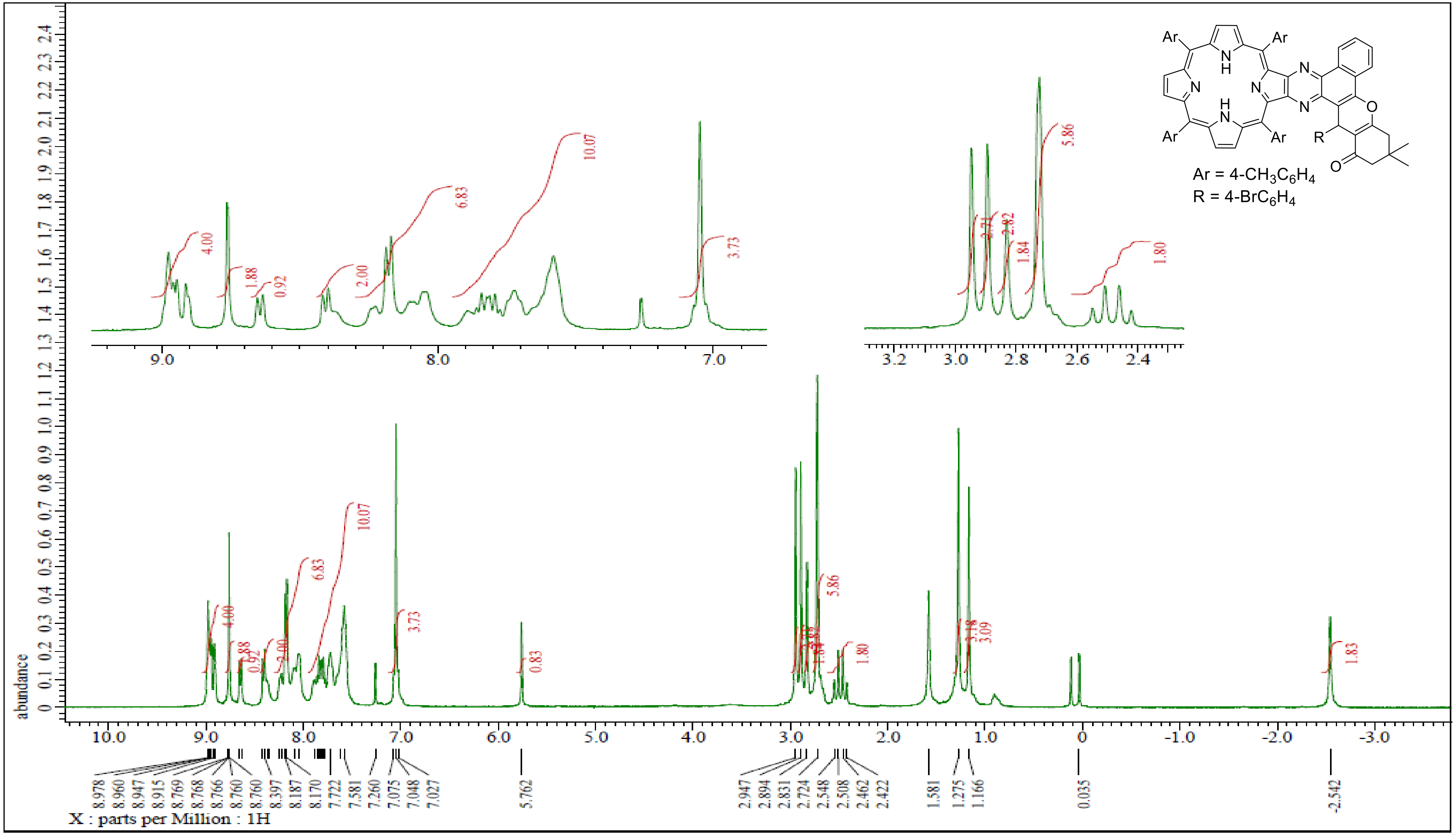

Figure 6. ^1H NMR spectrum of porphyrin **12** in $CDCl_3$.

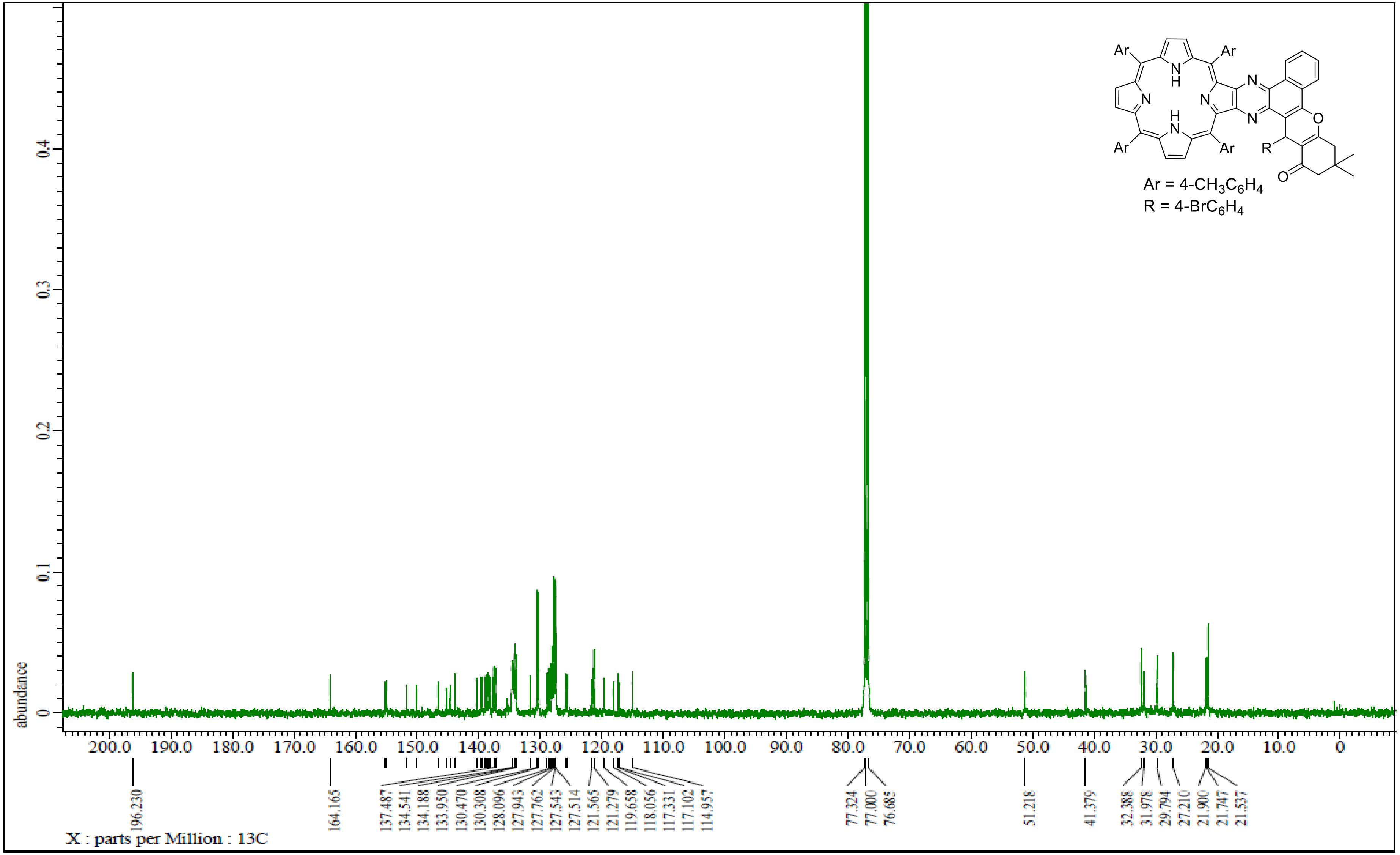

Figure 7. ^{13}C NMR spectrum of porphyrin **12** in $CDCl_3$.

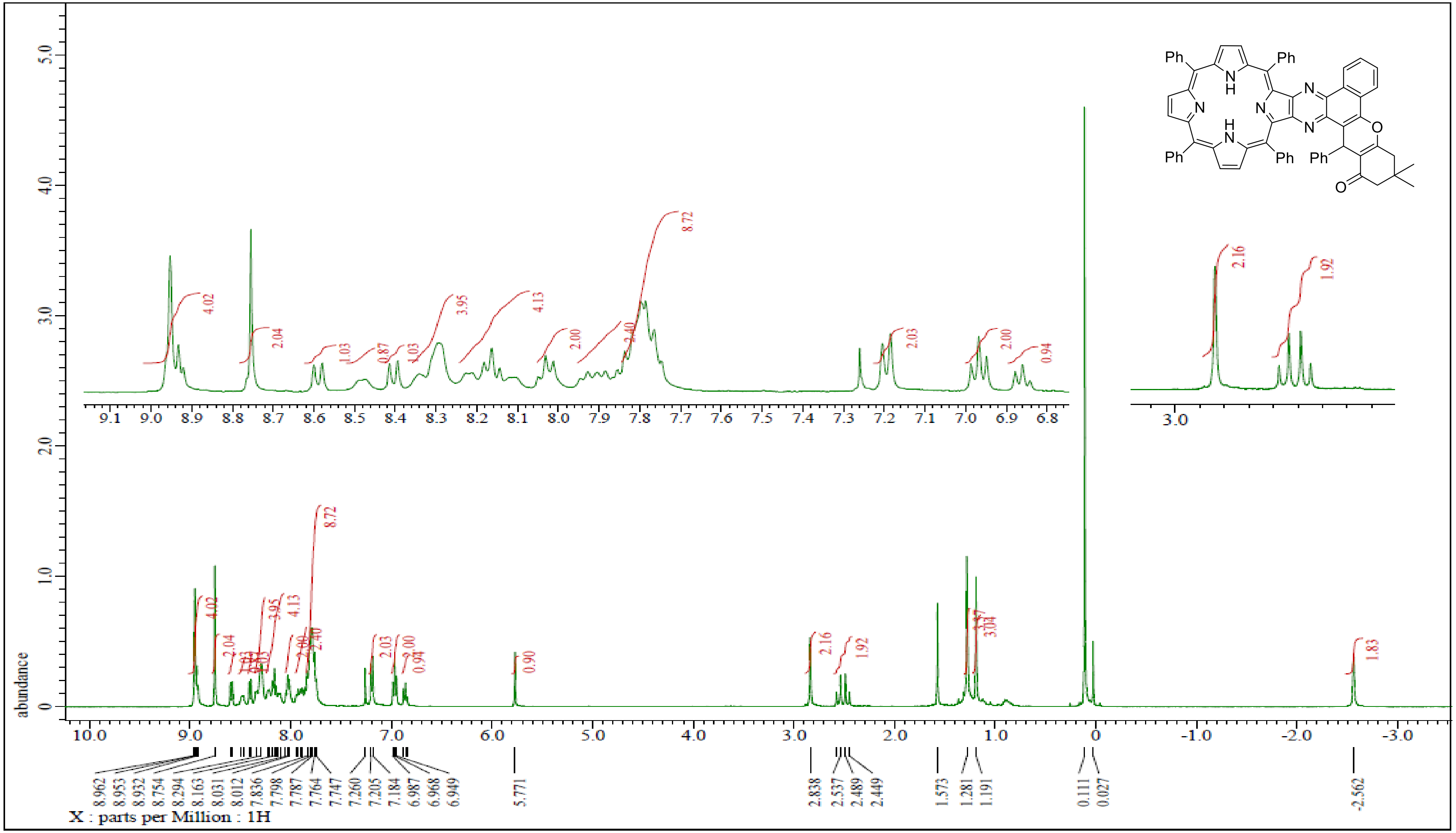

Figure 8. ^{1}H NMR spectrum of porphyrin **13** in $CDCl_3$.

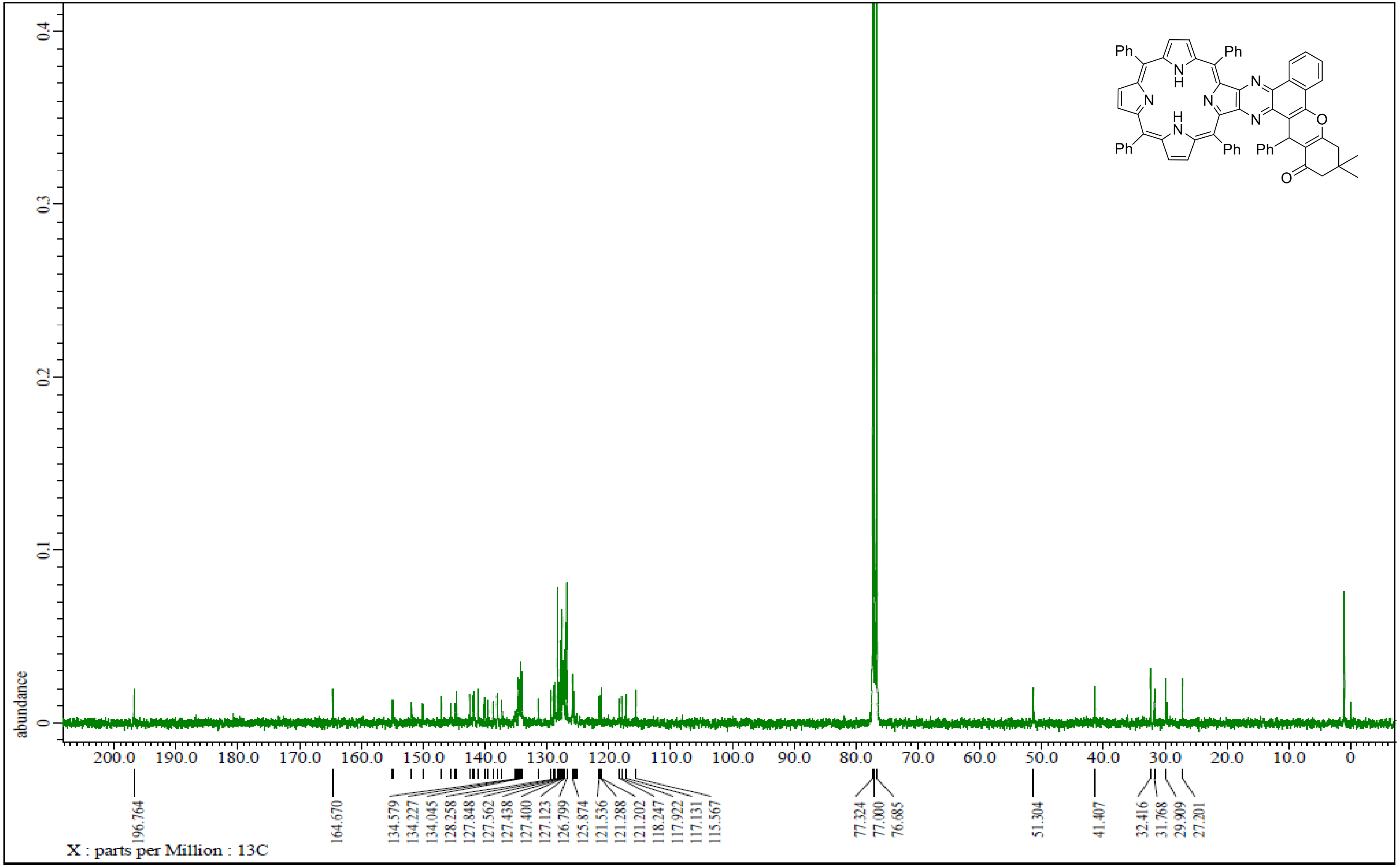

Figure 9. ^{13}C NMR spectrum of porphyrin **13** in $CDCl_3$.

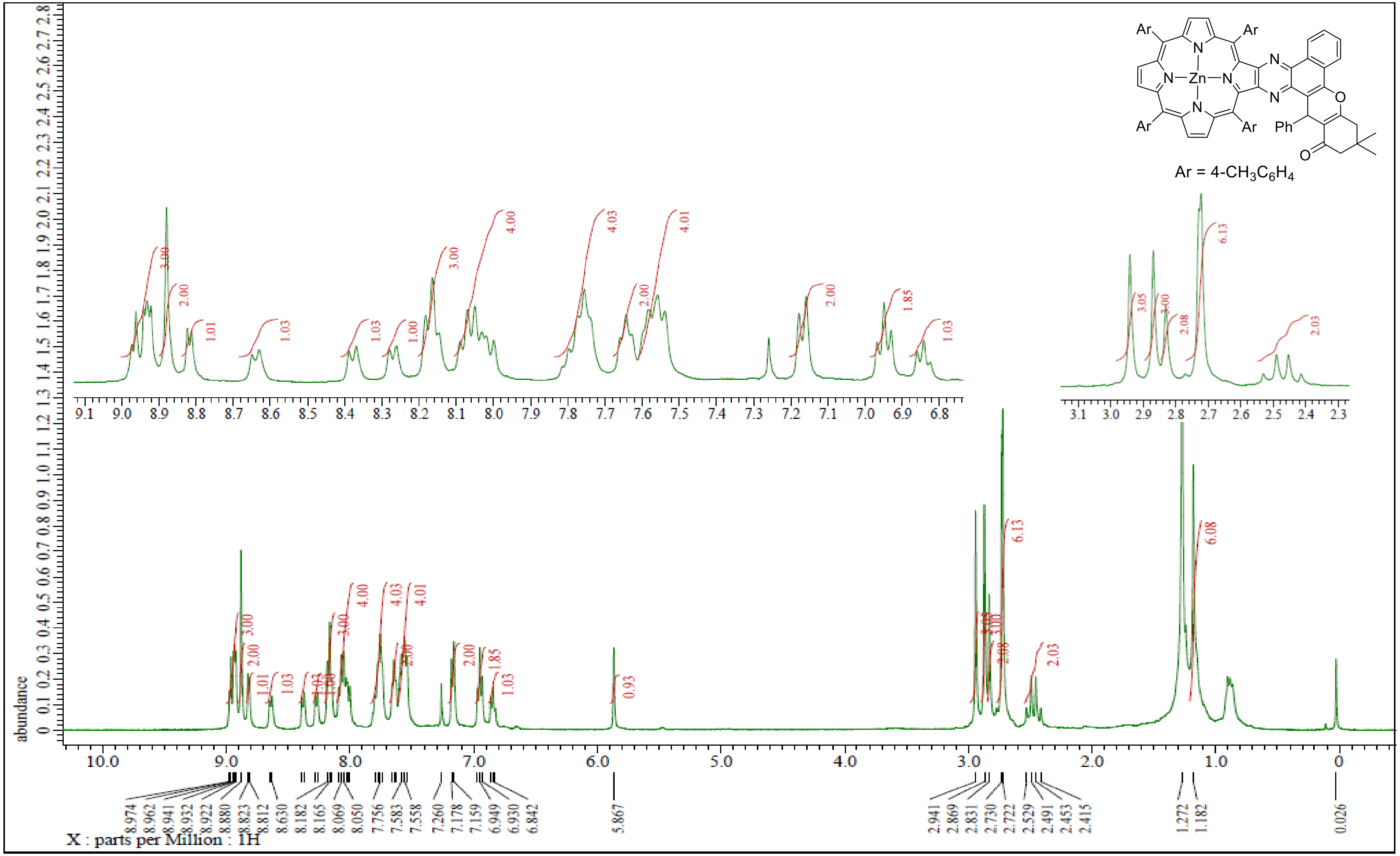

Figure 10. ^{1}H NMR spectrum of porphyrin **14** in $CDCl_3$.

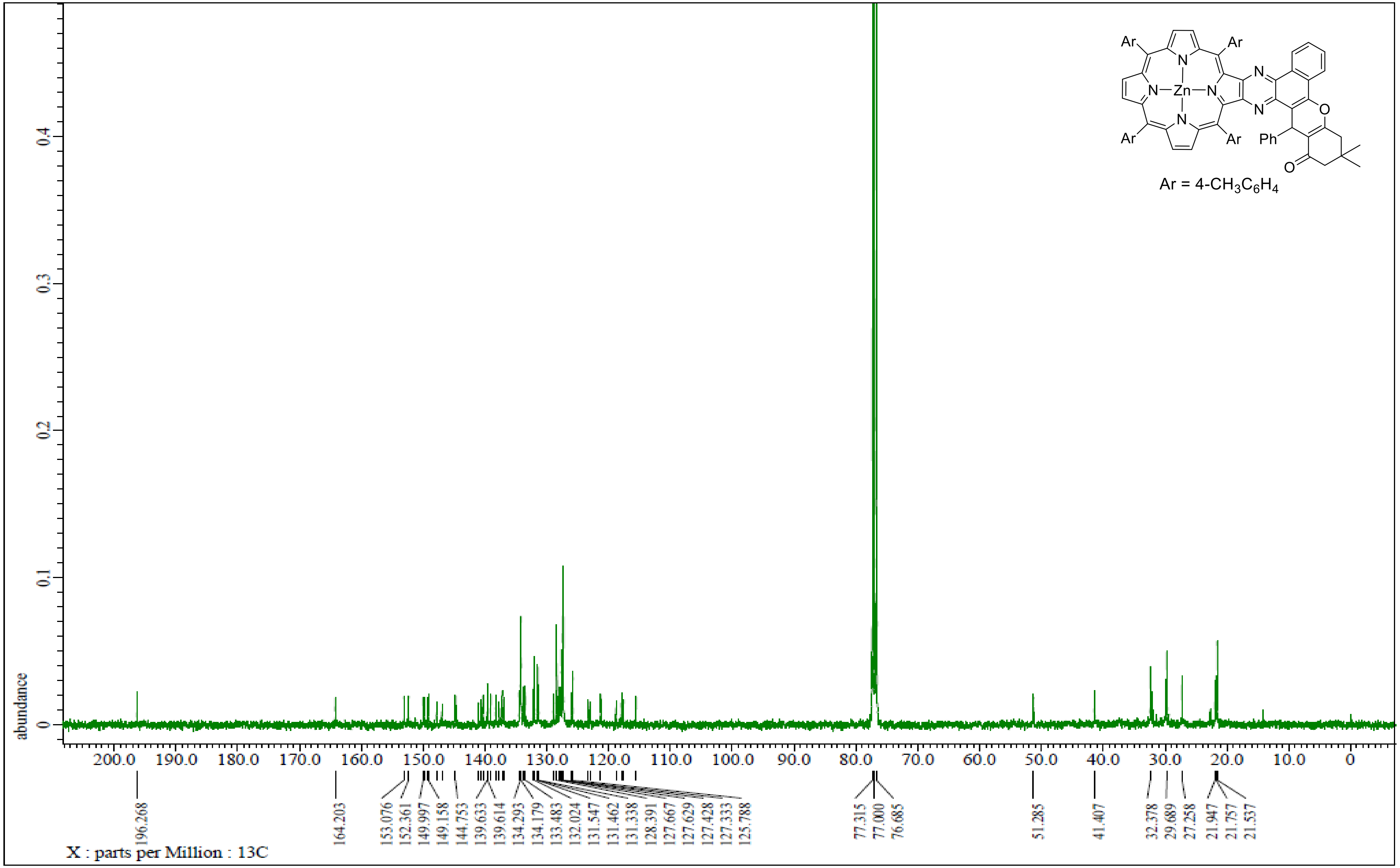

Figure 11. ^{13}C NMR spectrum of porphyrin **14** in $CDCl_3$.

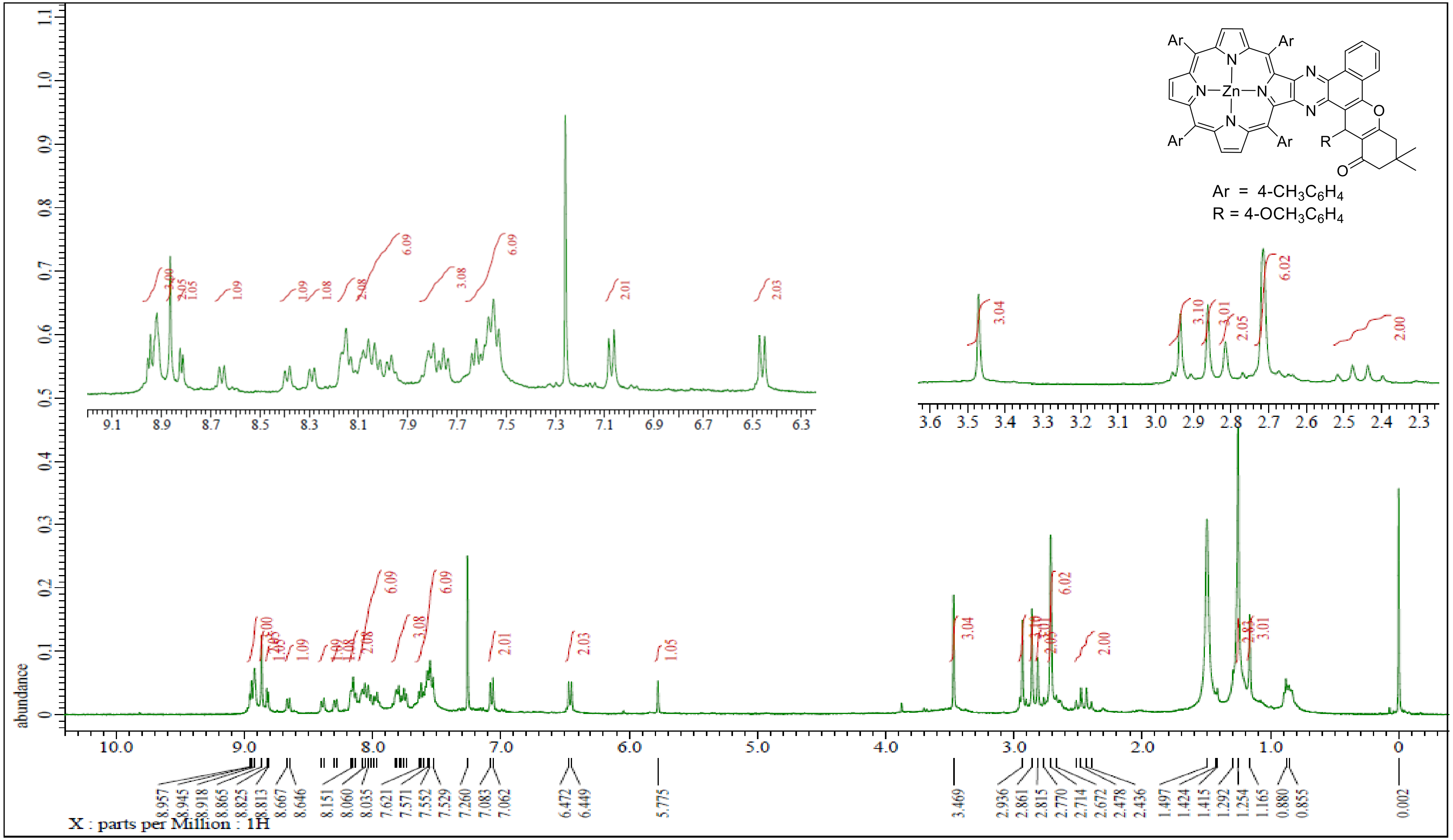

Figure 12. ^{1}H NMR spectrum of porphyrin **15** in $CDCl_3$.

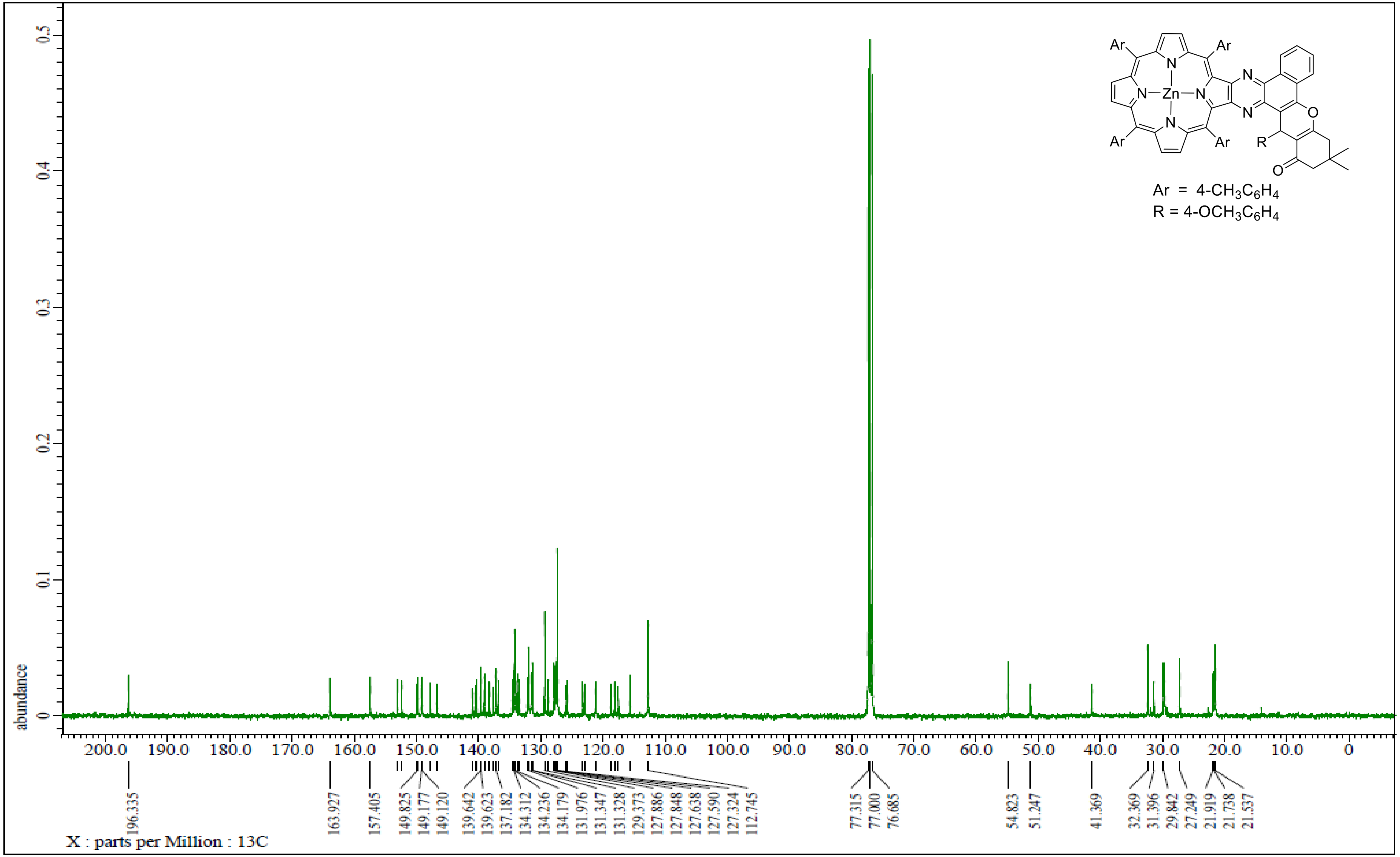

Figure 13. ^{13}C NMR spectrum of porphyrin **15** in $CDCl_3$.

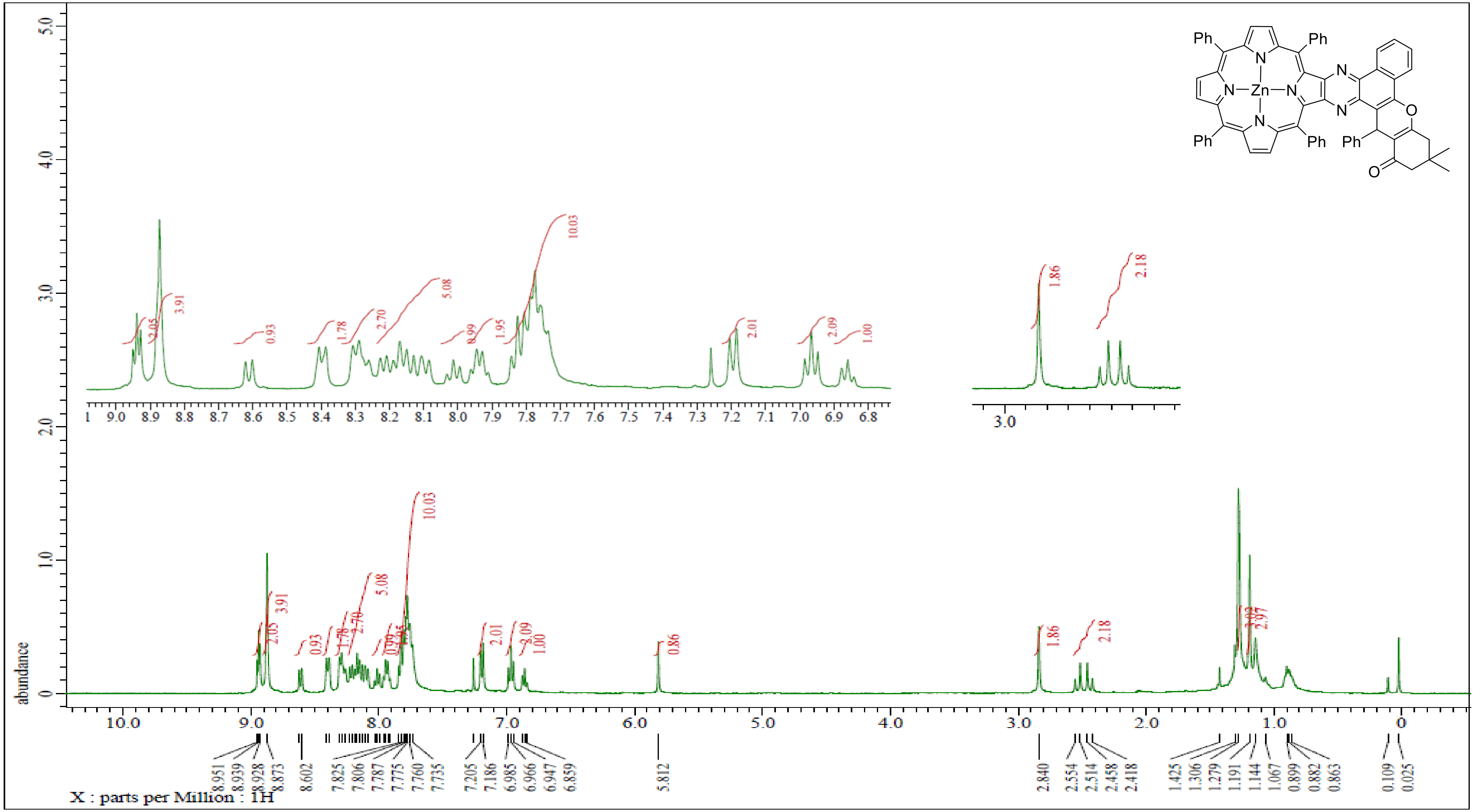

Figure 14. ^{1}H NMR spectrum of porphyrin **16** in $CDCl_3$.

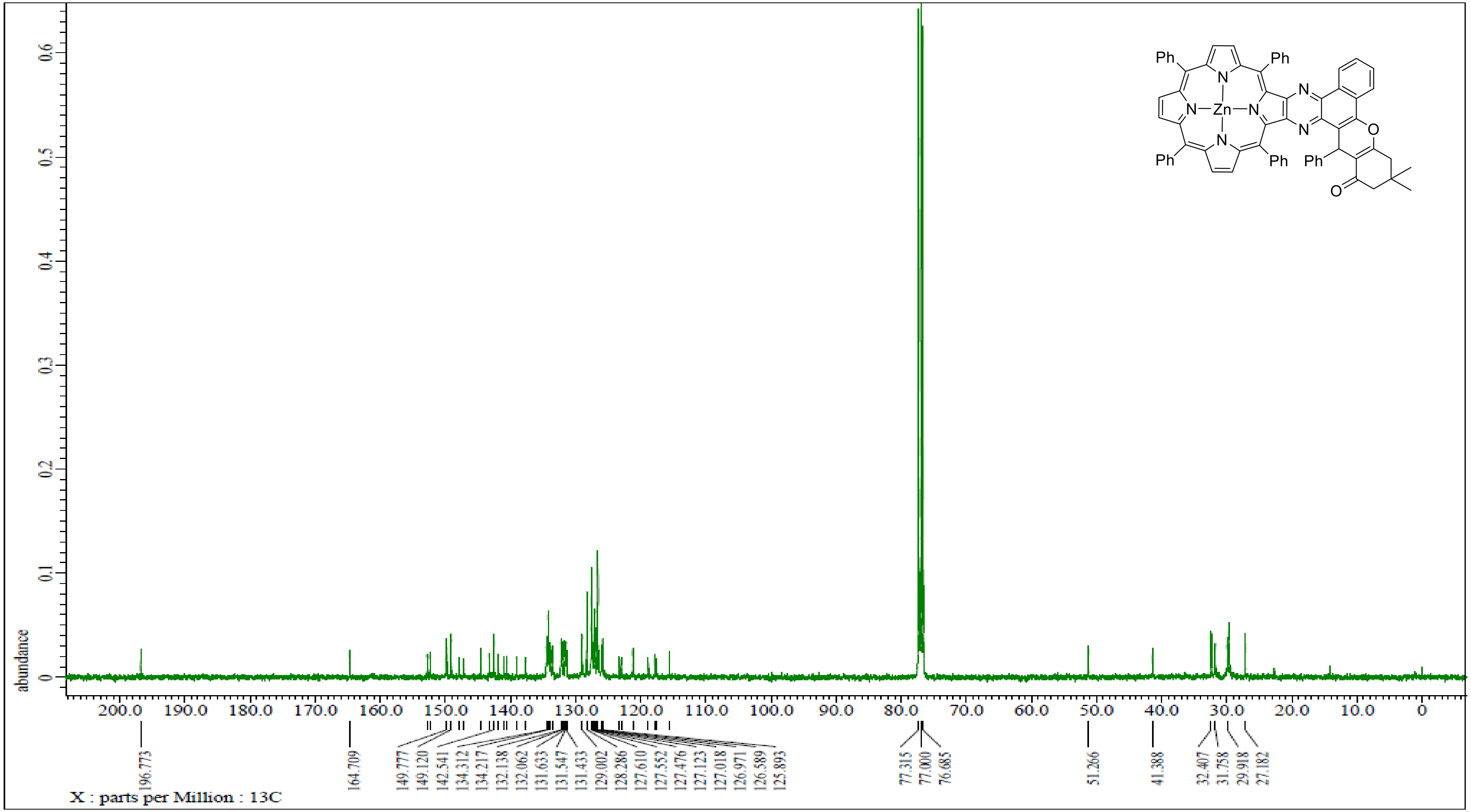

Figure 15. ^{13}C NMR spectrum of porphyrin **16** in $CDCl_3$.
